# Cap-adjacent 2′-*O*-ribose methylation of RNA in *C. elegans* is required for postembryonic growth and germline development in the presence of the decapping exonuclease EOL-1

**DOI:** 10.1093/nar/gkag355

**Published:** 2026-04-30

**Authors:** Eileen Clemens, Sarah Brivio, Mohammed Al-Khafaji, Peter Eijlers, Maheshika Kurukulasuriya, Irmgard U Haussmann, David MacLeod, Marius Wenzel, Berndt Müller, Matthias Soller, Jonathan Pettitt

**Affiliations:** School of Medicine, Medical Sciences and Nutrition, Institute of Medical Sciences, University of Aberdeen, Aberdeen AB25 2ZD, United Kingdom; School of Medicine, Medical Sciences and Nutrition, Institute of Medical Sciences, University of Aberdeen, Aberdeen AB25 2ZD, United Kingdom; School of Medicine, Medical Sciences and Nutrition, Institute of Medical Sciences, University of Aberdeen, Aberdeen AB25 2ZD, United Kingdom; School of Medicine, Medical Sciences and Nutrition, Institute of Medical Sciences, University of Aberdeen, Aberdeen AB25 2ZD, United Kingdom; School of Medicine, Medical Sciences and Nutrition, Institute of Medical Sciences, University of Aberdeen, Aberdeen AB25 2ZD, United Kingdom; Department of Life and Sports Sciences, School of Life Science and Health Sciences, Birmingham City University, Birmingham B15 3TN, United Kingdom; School of Medicine, Medical Sciences and Nutrition, Institute of Medical Sciences, University of Aberdeen, Aberdeen AB25 2ZD, United Kingdom; School of Biological Sciences, University of Aberdeen, Aberdeen AB24 2TX, United Kingdom; School of Medicine, Medical Sciences and Nutrition, Institute of Medical Sciences, University of Aberdeen, Aberdeen AB25 2ZD, United Kingdom; Division of Molecular and Cellular Function, School of Biological Sciences, Faculty of Biology, Medicine and Health, University of Manchester, Manchester M13 9PT, United Kingdom; School of Medicine, Medical Sciences and Nutrition, Institute of Medical Sciences, University of Aberdeen, Aberdeen AB25 2ZD, United Kingdom

## Abstract

Cap-adjacent 2′-*O*-ribose methylation (cOMe) of the first two transcribed nucleotides of RNA polymerase II transcripts is a conserved feature in many eukaryotes. In mammals, these modifications are key to a transcript surveillance system that regulates the interferon response, but the broader functions of cOMe remain poorly understood. To understand the role of cOMe in *Caenorhabditis elegans*, we functionally characterized the methyltransferases (CMTR-1 and CMTR-2) responsible for installing these modifications. These enzymes have distinct expression patterns, protein interaction partners, and loss-of-function phenotypes. Loss of CMTR-1 causes dramatic reductions in cOMe, impaired growth, and sterility. In contrast, animals lacking CMTR-2 are superficially wild-type, though CMTR-2 loss enhances the severity of the *cmtr-1* mutant phenotype. Depletion of CMTR-1 causes downregulation of transcripts associated with germline sex determination and upregulation of those involved in the intracellular pathogen response (IPR). We show that the absence of the decapping exonuclease, EOL-1, an IPR component, completely suppresses the sterility and growth defects caused by CMTR-1 loss, and reverses the associated steady-state transcript changes. Our work shows the physiological relevance of cOMe in protecting transcripts from decapping exonucleases, raising the possibility that cOMe plays a role in RNA-mediated immune surveillance beyond the vertebrates.

## Graphical Abstract

**Graphical Abstract:**
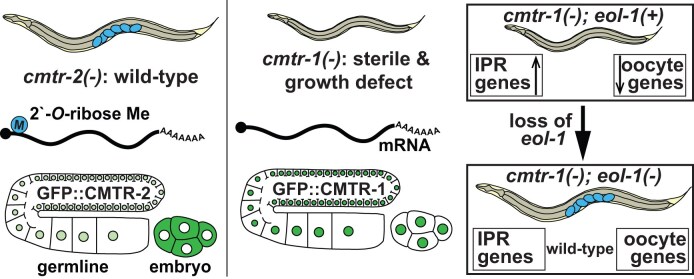

## Introduction

Cap-adjacent 2′-*O*-ribose methylation (cOMe) of RNA was first discovered in mammalian cells several decades ago [1–4]. Subsequent work showed that these modifications are found throughout the Metazoa, as well as in the viruses and protists that infect these organisms [5, 6]. In animals, the first two transcribed nucleotides can harbour cOMe, with methylation of the first transcribed nucleotide being designated ‘cap1’, and methylation of both first and second nucleotides referred to as ‘cap2’. Although these modifications are ubiquitous, their physiological significance remains incompletely understood.

The first defined and the best understood role for cOMe is in preventing activation of an RNA-based immune response [7–10]: an antiviral mechanism consisting of sensor proteins that recognize transcripts lacking cOMe, which brings about the activation of the interferon response. This has created strong selective pressure for viruses to evolve the means to ensure their transcripts are modified by cOMe [11]. However, this function does not explain the presence of cOMe in animals that lack the interferon pathway. It also does not explain the full significance of cOMe in mammals: loss of either of the two highly conserved methyltransferases, CMTR1 and CMTR2, responsible for cOMe in mice results in embryonic lethality without inducing the interferon pathway [12]; and there are multiple lines of evidence supporting diverse roles for cOMe, including the regulation of transcription, RNA splicing, and translation [13–18].

Studying cOMe in genetically tractable model organisms such as *Drosophila melanogaster* and *Caenorhabditis elegans* should allow us to understand the functional significance of cOMe from a whole-animal perspective. Both organisms have orthologues of mammalian CMTR1 and CMTR2 [6, 18]. However, detailed functional genetic analysis in *Drosophila* has been hampered by the relatively subtle phenotypic consequences of loss of cOMe. *Drosophila CMTr1*/*2* double mutants are viable, though they show specific behavioural and morphological defects consistent with defects in the spatial regulation of translation in neurons [18].

Previous studies have shown that subcellular mislocalization of *C. elegans* CMTR-1 can affect the expression of specific genes [19], and more recently, a hypomorphic *cmtr-1* mutation was recovered in a screen for mutants that showed elevated activation of an intestinal immune response. Loss of *cmtr-1* function correlated with increased resistance to infection with *Pseudomonas aeruginosa*, indicating a possible role for cOMe in the nematode immune response [20]. Here, we present a comprehensive analysis of CMTR function, showing that complete loss of cOMe in *C. elegans* leads to growth retardation and pronounced defects in germline development, with *cmtr-1* loss-of-function mutants conferring a fully penetrant sterile phenotype. Loss of cOMe leads to activation of a set of genes that constitute the intracellular pathogen response (IPR), an innate immunity and stress response that is activated by a range of exogenous and endogenous agents [21], raising the possibility that, as in mammals, there exists a mechanism to respond to transcripts that lack cOMe modifications. From a forward genetic screen for suppressors of *cmtr-1* loss-of-function, we identified the decapping exonuclease EOL-1, showing that this enzyme is a major component of the cellular response to transcripts lacking cOMe. Our work suggests evolutionary continuity in the role of cOMe in RNA immune surveillance and provides physiological support for the role of this modification in protecting transcripts from degradation by the DXO/Rai decapping exonuclease family.

## Materials and methods

### Nematode strains

*Caenorhabditis elegans* strains were grown using standard culture conditions [22] at 20°C unless otherwise indicated. N2 (Bristol) was used as the wild type. The following previously described strains were used in this study: HML1012 [23], CGC45, and CGC66. Strains generated in the current study are described in Supplementary Table S1.

The *cmtr-1* deletion allele, *syb3613*, was generated by Fujian SunyBiotech Co. Ltd, and obtained as strain PHX3613. The *cmtr-2* deletion allele, *tm4453*, was obtained from the Mitani Lab as strain FX4453. PE1000 was generated from FX4453 by outcrossing six times against N2. Fluorescent-tag/degron knock-in strains were created as described previously [23–25]. The CMTR-1(K244A) mutation was generated through oligonucleotide-templated repair of CRISPR/Cas9-induced double-strand breaks, using the previously described *dpy-10*-based co-conversion strategy [26]. CRISPR alleles of *eol-1* were made using the same *dpy-10*-based approach, but the injections were performed using PE1176 animals and the resultant F1 progeny grown on 5-Ph-IAA-containing plates, selecting for suppression of the sterile phenotype. Alleles were confirmed by Sanger sequencing (Eurofins Genomics). Oligonucleotides used to verify the allelic status of genes, generate guide RNA constructs, and produce homologous repair templates are given in Supplementary Table S1.

### Transgenic rescuing assays

The wild-type *cmtr-1* complementary DNA (cDNA) was amplified from total RNA using primers cmcDNAFo and cmcDNARev (Supplementary Table S1). The plasmid backbone, which contains the *rps-0* promoter and *unc-54* 3′ UTR, was amplified using primers prpo-u54Fwd and prpo-u54Re (Supplementary Table S1), and the *cmtr-1* cDNA cloned between the *rps-0* promoter and *unc-54* 3′ UTR using the NEBuilder HiFi DNA Assembly kit (NEB) to create plasmid pSB2. The plasmid was verified by Sanger sequencing (Eurofins Genomics). PE1033 worms were co-injected with pSB2 and a plasmid containing *myo-2p::tdTomato* [27]. Transgenic lines were established as previously described [28, 29]. The rescue assay was performed by picking non-tdTomato offspring as L1/L2 larvae to separate plates and scoring their ability to reach fertile adulthood.

The wild-type *eol-1* locus was amplified from N2 genomic DNA using primers eol1fo1 and eol1re1 (Supplementary Table S1). The resultant polymerase chain reaction (PCR) product was verified by Sanger sequencing (Eurofins Genomics) and shown to be ~2 kb larger than that predicted from the N2 reference genome assembly, but identical to the recently produced CGC1 reference genome [30], revealing the presence of an additional tandemly duplicated *eol-1* paralogue (herein referred to as *eol-1* paralogue B). The *eol-1* amplicon (25 ng/µl) was co-injected into PE1234 worms (*cshIs140[rps-28p::TIR1(F79G)(fe156)::T2A::mCherry::his-11 + Cbr-unc-119(+)] cmtr-1(fe148[mNG^AID::cmtr-1]) II; eol-1(fe153) V*) with *myo-2p::tdTomato* (10 ng/µl) and pUC18 (180 ng/µl). Transgenic animals were identified based on pharyngeal tdTomato expression.

### Fluorescent microscopy

Worms were mounted in 5 µl M9 supplemented with 10 mM sodium azide on 5% agar pads. Images were obtained using either a Zeiss Axioplan 2, equipped with a Hamamatsu Orca ER camera, or a Zeiss Imager M2 upright microscope, equipped with a Hamamatsu Flash 4 LT camera.

### Western blot

Western blots were performed as described previously [31] using lysates from 300 to 500 adult/L4 larvae, or 5000 L1 larvae per well. To detect embryonic expression of CMTR-1/-2 in embryos, 40 µg of total protein from embryonic extracts was loaded per gel lane. For detecting larval expression, GFP-tagged proteins were detected with mouse anti-GFP antibodies (Roche 11814460001), mNeonGreen-tagged proteins with rabbit anti-mNeonGreen antibodies (Proteintech, 29523-1-AP), and GAPDH using mouse anti-GAPDH antibodies (Invitrogen AM4300). Secondary antibodies used were anti-mouse or anti-rabbit HRP-conjugated IgG antibody (Cell Signalling Technology 7074 and 7076). To detect proteins in embryonic extracts, GFP-tag polyclonal antibody (Proteintech, 50430-2-AP) (1:1000 dilution) was used together with the above anti-rabbit secondary (1:3000 dilution). Blots were also stained with amido black and quantified using either Fiji [32]or ImageQuant (Cytiva) software [31].

### Preparation of *C. elegans* embryo extracts

*C. elegans* embryo extracts were prepared essentially as described previously [27]. Extracts were treated with RNase as described previously [27].

### Cap-methylation status analysis of mRNA

Total RNA was extracted with Trizol (Sigma) according to the manufacturer’s description, using 20 µg glycogen (Roche) for precipitation. Poly(A) messenger RNA (mRNA) was prepared using the NEBNext® Poly(A) mRNA Magnetic Isolation Module (NEB) by double oligo dT selection according to the manufacturer’s instructions.

For the analysis of 5′ cap structures, 5 µg total RNA was used for decapping by yDcpS in 20 µl for 1 h at 37°C according to the manufacturer’s instruction (NEB). The RNA was extracted by phenol/CHCl_3_ and ethanol precipitated in the presence of glycogen. The RNA was then labelled in a total volume of 20 µl containing 2 µl capping buffer (NEB), 1 µl SAM (2 mM), 0.25 µl ^32^P-αGTP (3000 Ci/mmol, 6.6 µM; Hartmann Analytics), 0.5 µl RNase Protector (Roche), and 0.5 µl capping enzyme (NEB) by incubation for 1 h at 37°C. The volume was then increased to 50 µl with water, and poly(A) RNA selected as described earlier. The RNA was then digested on the beads in 5 µl using 0.5 µl NEB buffer 3 and 0.5 µl RNase I for 2 h, and then 10 µl gel loading buffer was added [98% deionized formamide,10 mM ethylenediaminetetraacetic acid (EDTA), 0.025% xylene cyanol FF and 0.025% bromophenol blue]; products were analysed on 22% denaturing polyacrylamide gels (National Diagnostics) and pre-run for 2 h. Gels were soaked in 20% PEG400, 10% acetic acid, and 40% methanol for 10 min and then dried on a Whatman 3MM paper. Dried gels were then exposed to a storage phosphor screen (Bio-Rad) and scanned by a Molecular Imager FX in combination with QuantityOne software (Bio-Rad). Markers were prepared as described previously [6, 18].

For the analysis of the first nucleotide in mRNA, 5 µg poly(A) mRNA was purified as described earlier and 5 µl incubated with terminator nuclease (Epicenter), according to the manufacturer’s instructions, to remove ribosomal RNA, followed by phenol/CHCl_3_ and ethanol precipitation in the presence of glycogen (Roche). The mRNA was then decapped using RppH (NEB) and dephosphorylated by Antarctic phosphatase (NEB) in NEB buffer 2 supplemented with 0.1 mM ZnCl_2_ in 20 µl. Then, the RNA was extracted by phenol/CHCl_3_ and precipitated in the presence of glycogen. The 5′-ends of dephosphorylated mRNAs were then labelled using 10 units of T4 PNK (NEB) and 0.25 µl ^32^P-γATP (6000 Ci/mmol, 12.5 µM; Hartmann Analytics). The labelled RNA was precipitated and resuspended in 10 µl of 50 mM sodium acetate buffer (pH 5.5) and digested with nuclease P1 (SIGMA) for 2 h at 37°C. Two microliters of each sample was loaded on cellulose F TLC plates (20 × 20 cm; Merck) and run in a solvent system of isobutyric acid:0.5 M NH_4_OH (5:3, v/v, solvent A), as first dimension, and isopropanol:HCl:water (70:15:15, v/v/v, solvent B) or sodium phosphate, 0.1 M (pH 6.8), ammonium sulfate, n-propanol (100:60:2, v/w/v, solvent C), as the second dimension. TLCs were repeated from biological replicates. The identity of the nucleotide spots was determined as described [33, 34]. TLCs were exposed to a storage phosphorscreen (Bio-Rad) and scanned by a Molecular Imager FX in combination with QuantityOne software (Bio-Rad).

### Monitoring SL1 *trans*-splicing using quantitative PCR

RNA was isolated from embryonic extracts prepared from PE1176 and PE1220 embryos, treated for 4 h with either 50 µM 5-Ph-IAA-AM in DMSO or control treated in DMSO by extraction with trizol, and then purified using the PureLink RNA Mini kit (Life Technologies), with modifications for trizol-treated samples and DNase treatment, as described by the manufacturer. Reverse transcription and analysis by qPCR were done as described [27, 35]. Analyses were done as three technical replicates. Shown are Δ*C*_T_ values derived for each replicate [36].

### *In vitro* spliced leader *trans*-splicing assays

For extract preparation, PE1176 and PE1220 animals were grown in liquid culture, and embryos were isolated and treated with 50 µM 5-PH-IAA-AM or control-treated with DMSO as described [27], except that treatment was for 4 h. Extracts were then prepared as described [27] and stored at −80°C. The synthetic SL *trans*-splicing substrate, consisting of *rps-3* genomic DNA containing 404 bp of 5′ UTR sequence (including 382 bp of outron sequence upstream of the known SL1 *trans*-splice site) and extending 266 base pairs downstream from the ATG translation start site, was amplified from *C. elegans* genomic DNA using primers *rps-3* genFwd and *rps-3* genRev (Supplementary Table S1). The resulting amplicon was inserted into pBlueScript KS(-) cleaved with XbaI using NEB HIFI gene assembly to produce pBS-*rps-3*. The sequence was confirmed by Sanger sequencing (Eurofins Genomics). pBS-*rps-3* DNA linearized with EcoRI was transcribed with T7 RNA polymerase using the MEGAscript® kit (Invitrogen). This produces a synthetic *rps-3* RNA with a 3′-end derived from pBlueScript KS(-) that serves as a primer binding site in PCR. RNA was extracted using phenol/chloroform, concentrated by ethanol precipitation, resuspended in RNase-free water, and purified using MicroSpin G-25 columns (Cytiva). RNA concentration was determined by measuring absorption at 260 nm assuming 1 AU = 40 µg/ml.

*In vitro* SL1 *trans*-splicing reactions were done essentially as described [37]. Fifteen microliters reactions were done in 10 mM Tris–HCl (pH 8.0), 60 mM KCl, 4 mM MgCl_2_, 2 mM ATP, 20 mM creatine phosphate, 50 μg/ml creatine kinase, 2 mM DTT, 3% PEG 8000, 0.25 mM EDTA, 5% glycerol, 0.5 mM PMSF, 2 U/μl RNaseOUT, 8 µg/μl embryonic extract, and 2.5 ng/μl synthetic *rps-3* mRNA. Control reactions were performed without addition of ATP, creatine phosphate, and creatine kinase. Incubation was at 15°C for 2 h.

For analysis by one-step reverse transcriptase–polymerase chain reaction (RT-PCR), reactions were diluted 1:80 in RNase-free MilliQ water and aliquots were then used for one-step RT-PCR using the Luna® Universal One-Step RT-qPCR kit (NEB) according to the manufacturer’s instructions; primers are given in Supplementary Table S1. Reactions were done with 1 or 2 µl diluted reactions and analysed by agarose gel electrophoresis, or quantified using a LightCycler 480 (Roche), with software release 1.5.1.62 Sp3. qPCR data were analysed using the ∆C_T_ method [36]. Products P1, P2, and P3 were identified by cloning into pGEM-T Easy (Promega) followed by Sanger sequencing (Eurofins Genomics).

### Protein immunoprecipitation

Immunoprecipitations of GFP-tagged proteins were performed in triplicates or quadruplicates using anti-GFP nanobody-coupled agarose beads and control agarose beads (GFP-Trap and control agarose beads, Chromotek GmbH), essentially as described previously [27].

### Protein analysis by LC-MS/MS and differential protein expression analysis

Proteomic analysis by mass spectrometry was done at the Aberdeen Proteomics unit as previously described [27, 31]. MaxQuant version 1.6.5.0 [38] was used to process the raw data files with the *C. elegans* reference proteome UP000001940 downloaded on the 24th of August 2021 as described [27], except that the feature ‘match between runs’ was applied. The MaxQuant protein group files were further analysed using Perseus version 1.6.5.0 as described [27]. Graphs were drawn in GraphPad Prism.

### RNA extraction and RNA-seq

Prior to RNA extraction, the worms were treated with 5-Ph-IAA when they reached the L3 larval stage. Therefore, synchronized L1s were grown in liquid culture for 22.5 h until they started moulting into larval stage 3. They were then divided into two and plated on control plates or 5-Ph-IAA plates, respectively, and cultured for 16 h, at which point the animals had started moulting into the L4 larval stage. Worms were washed off NGM plates using M9 and washed three times by centrifugation at 749 × *g* for 2 min at 4°C. Subsequently, 1 ml of Trizol (TRI Reagent, T9424, Sigma–Aldrich) was added per 50 µl of worm pellet, and samples frozen in liquid nitrogen. Samples were thawed at 37°C and re-frozen four times. They were then vortexed for 30 s, left to rest for 30 s, and the vortex/rest process repeated for a further three times. Phase separation by addition of chloroform and recovery of RNA was done as described by the manufacturer. The aqueous fraction was transferred to a low DNA-binding-tube and diluted 1:1 with 70% ethanol. RNA was purified using PureLink RNA Mini Kit (Thermo Fisher Scientific), using the on-column DNase-treatment option. Three libraries per treatment group were sequenced on an Illumina NovaSeq instrument, generating 2 × 150 bp paired-end reads (Novogene UK Ltd).

### Differential gene expression analysis

The quality of the raw reads was inspected in FASTQC 0.11.9 [https://www.bioinformatics.babraham.ac.uk/projects/fastqc/] and MULTIQC 1.12 [39], and bases with a Phred score below 20 were trimmed using TRIMGALORE 0.6.6 [https://www.bioinformatics.babraham.ac.uk/projects/trim_galore/]. The trimmed reads were then aligned to the *C. elegans* WB235 reference genome using HISAT2 2.2.0 [40]. Alignments were processed using SAMTOOLS 1.14 [41]. Gene-level read counts were obtained using FEATURECOUNTS 2.0.2 [42], quantifying against exon annotations and assigning fractional counts to all alignment locations of multi-mapping reads.

The read counts were analysed in DESeq2 1.42 [43], identifying differentially expressed genes (DEGs) with an FDR [44] significance threshold of 0.05 and shrinking fold changes at low-count genes using the *apeglm* method [45]. Fold changes of predefined sets of genes (IPR genes and germline sex determination genes) were compared against all other genes using two-sample Welch *t*-tests. Significantly DEGs were examined for overrepresented Gene Ontology (GO) terms (biological process ontology) using CLUSTERPROFILER 4.10.0 [46] with the *org.Ce.eg.db* 3.18.0 database (June 2024). Significantly overrepresented GO terms were selected based on a *q*-value threshold of 0.05. The GO results were simplified by removing terms at graph levels 1–3, clustering GO terms semantically with the Wang method at a similarity cutoff of 0.7 [47], and retaining for each cluster the single GO term with the smallest *P*-value.

### Data analysis using RStudio

RStudio was used for data analysis and graph generation (http://www.rstudio.com/). The following R packages were used: ggplot2 [48], dplyr (https://dplyr.tidyverse.org/), carData/car (10.32614/CRAN.package.car), multcomp (10.32614/CRAN.package.multcomp), ggpubr (https://rpkgs.datanovia.com/ggpubr/), rstatix (https://rpkgs.datanovia.com/rstatix/), tibble (https://tibble.tidyverse.org/), and RcolorBrewer (10.32614/CRAN.package.RColorBrewer).

### Screen for suppressors of *cmtr-1* loss-of-function

PE1176 worms were subjected to a standard ethyl methylsulfonate (EMS) mutagenesis. Thirty F1 cultures were established by subjecting the mutagenized parental animals to alkaline hypochlorite treatment and plating 500 F1 animals per 9 cm plate. The mutagenized animals and their F1 progeny were propagated on standard NGM agar plates. The F2 embryos from alkaline hypochlorite-treated F1s were plated onto 9 cm NGM plates supplemented with 1 µM 5-Ph-IAA. Suppressor mutants, identified on the basis of presence of eggs in the uterus of adult F2s, were picked to establish lines, taking care to only maintain one line for each F1 lineage.

### Genetic analysis of suppressor mutants

We reasoned that our screen would generate two classes of fertile mutants: those that were *bona fide* extragenic suppressors of mNG^AID::CMTR-1 knockdown and loss-of-function mutants in the *TIR1* transgene, which would lead to 5-Ph-IAA-independent expression of mNG^AID::CMTR-1. We thus screened each suppressor line for the presence of nuclear mNeonGreen fluorescence when grown on media containing 5-Ph-IAA. Mutant lines that showed mNeonGreen fluorescence in the presence 5-Ph-IAA were assumed to be *TIR1* mutants; the *TIR1* coding region for a subset of these was sequenced to confirm this assumption. Amplicons generated from single-worm PCRs using primer pairs TIR1Fwd + TIR1Rev1 and TIR1fo1 + TIR1re2 were sequenced using the same oligos to prime Sanger sequencing reactions (Eurofins Genomics).

Mutants that lacked nuclear mNeonGreen fluorescence in the presence of 5-Ph-IAA were assumed to be genuine epistatic suppressors and were backcrossed with non-mutagenized PE1176. The resultant strains were crossed into the *cmtr-1(syb3613)* background to determine the ability of the suppressor mutations to suppress the sterile phenotype of the *cmtr-1* null allele.

### Sib-selection and DNA preparation

Two suppressor strains, PE1237 (*fe152*) and PE1245 (*fe154*), were subject to a sibling subtraction/whole-genome sequencing method adapted from a previously reported method [49]. Male PE1053 worms were crossed with hermaphrodites from PE1237/1245. F1 progeny carrying the *feEx342* transgenic array were identified on the basis of pharyngeal tdTomato expression, picked to separate plates, and allowed to segregate F2 progeny. Two hundred *feEx342* transgenic F2s were picked singly to separate 35 mm plates and allowed to propagate. Homozygous suppressor lines (Sup) were identified based on the presence of fertile non-tdTomato F3/F4 worms, and the absence of non-tdTomato ‘scrawny’ F3/F4 worms, the latter being observed on plates whose frequency suggested that these were broods of suppressor heterozygotes. Lines that failed to inherit suppressor mutations (Nonsup) were identified on the basis of the complete absence of fertile non-tdTomato F3/F4 worms. Contaminated plates were discarded. Worms were washed off plates, and Sup/Nonsup cultures pooled as outlined previously [50]. The number of 35 mm F2 plates used to generate the Sup and Nonsup pools were as follows: *fe152* Sup = 16; *fe152* Nonsup = 19; *fe154* Sup = 19; *fe154* Nonsup = 19. Genomic DNA was prepared using the Qiagen Puregene Tissue Kit (Qiagen, 158 063) as described previously [50]. One library per pool was sequenced on an Illumina NovaSeq X Plus instrument, generating 2 × 150bp paired-end reads (Novogene UK Ltd).

### Variant detection

Quality control of the raw reads was carried out using FASTQC 0.11.9 (https://www.bioinformatics.babraham.ac.uk/projects/fastqc/) and MULTIQC 1.12 [39]. Adaptor removal and trimming of bases with a Phred score below 20 were performed using TRIMGALORE 0.6.6 (https://www.bioinformatics.babraham.ac.uk/projects/trim_galore/). The trimmed reads were then aligned to the *C. elegans* WB235 reference genome using BWA MEM 0.17.17 [51]. Variant detection was done for all libraries together in a single run of FREEBAYES 1.3.6 [52], and the resultant VCF files were filtered and manipulated using BCFTOOLS 1.14 [53]. We considered biallelic SNPs with experiment-wide genotyping quality scores of at least 20, and at least 10 reads in each library. We retained single nucleotide polymorphisms (SNPs) fixed for the alternative allele (>90% of read depth) in Sup libraries and fixed for the reference allele (>90% of read depth) in Nonsup libraries. SNPEFF 4.3t [54] was then used to functionally annotate the candidate SNPs identified by this filtering strategy.

## Results

### CMTR-1 is required for oocyte cell fate

**Figure 1.**
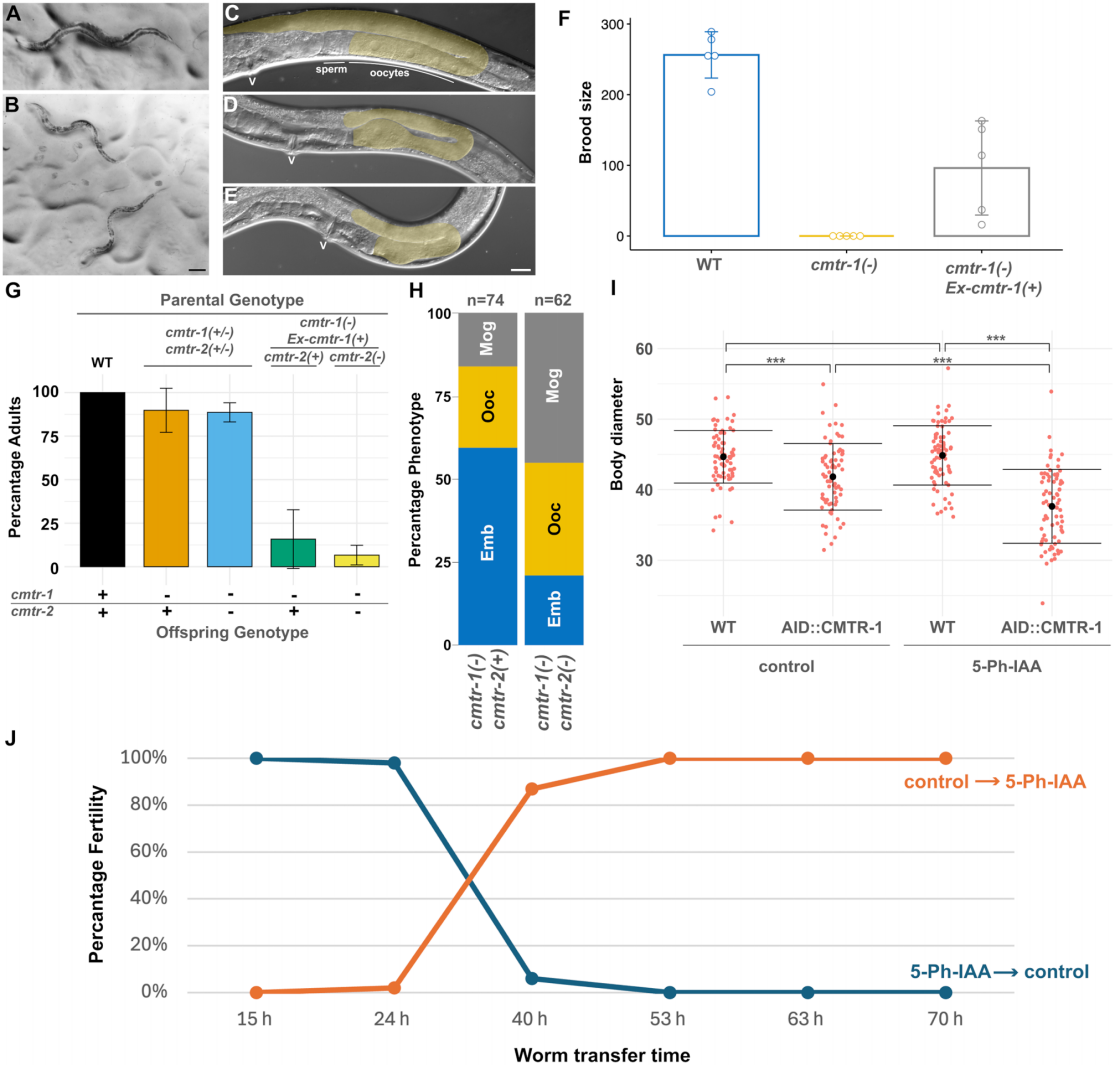
Loss of *cmtr-1* function results in defects in germline development and body size. Representative *cmtr-1(syb3613)/+* heterozygote (**A**), compared to sibling *cmtr-1(syb3613)* homozygotes (**B**). Wild-type young adult hermaphrodite (**C**), showing posterior gonad arm (gold overlay), compared to the same regions of *cmtr-1(syb3613)* homozygotes, one of which shows a single defective oocyte (**D**), and the other has a fully masculinized germline that has only produced sperm (**E**). Position of the vulva is indicated by ‘v’. Scale bars represent 100 µm (A, B) and 20 µm (C–E). (**F**) Transgenic expression of *cmtr-1* cDNA under the control of the *rps-0* promoter (*Ex-cmtr-1(+)*) rescues the fertility defects of *cmtr-1(syb3613)* mutants (*cmtr-1(-)*). (**G**) Comparison of the number of worms reaching adulthood 72 h after hatching (*n* > 61 for each experiment). Mutant offspring (*cmtr-1(-); cmtr-2(+)* or *cmtr-1(-); cmtr-2(-)*) of transgenic parents (transgene is indicated ‘*Ex-cmtr-1(+)*’) are slower-growing compared to genetically identical offspring of heterozygous parents. (**H**) Loss of *cmtr-2* increases the severity of the *cmtr-1* loss-of-function germline defects. Histograms show the distribution of the three germline defect classifications caused by loss of *cmtr-1* function: production of a few arrested embryos (Emb, blue); abortive oocyte production (Ooc, gold); and masculinized germline (Mog, grey). (**I**) Worm diameter, measured at the mid-body, for worms homozygous for the *mNG^AID::cmtr-1* allele subject to control and 5-Ph-IAA treatment. Two-way analysis of variance and a Tukey post hoc test were used to determine *P*-values; * < .05, ** < .01, *** < .001, *n* = 75 animals per group. (**J**) ‘Auxin-shift’ experiment to determine when CMTR-1 function is required for fertility. Starved L1 larvae (*n* = 50) were placed on either 5-Ph-IAA or control plates for the indicated time period, before being transferred to the alternative plate type (i.e. those on 5-Ph-IAA plates were moved to control plates, and vice versa). The worms were then left on the second plate, allowed to reach adulthood, and scored for fertility.

To determine the role of cOMe in *C. elegans*, we studied the phenotypes of animals lacking one or both CMTR proteins. We obtained a deletion allele, *syb3613*, of *cmtr-1* (Fujian SunyBiotech Co. Ltd), which removes the methyltransferase domain and is thus a predicted null allele. We also studied a deletion allele of *cmtr-2, tm4453*, that deletes key residues in the methyltransferase domain [6]. While *cmtr-1(syb3613)* homozygotes were able to reach adulthood, they were 100% sterile and were thinner than similarly staged *syb3613* heterozygotes (Fig. 1A and B).

We found that the sterility was due to defects in the formation of oocytes, which ranged from a completely masculinized germline that produced only sperm, through to animals that were able to produce only a few embryos that failed to develop (Fig. 1D, E, and H). In all cases, *cmtr-1(syb3613)* homozygotes showed much smaller germlines than wild-type worms, something also observed in mutants that cause masculinized hermaphrodite germlines [55].

We confirmed that the defects observed were due to loss of *cmtr-1* function by demonstrating that an extrachromosomal transgene consisting of the *cmtr-1* cDNA expressed under the control of the *rps-0* promoter rescued the germline sterility defects (Fig. 1F and G). As part of this analysis, we found that offspring that failed to inherit the extrachromosomal transgene generally showed a more severe phenotype than the offspring of heterozygous mothers, displaying more severe growth defects (Fig. 1G). Since extrachromosomal transgenes are generally silenced in the germline [56], this likely indicates that there is a significant *cmtr-1* maternal contribution, which is absent from the non-transgenic offspring of transgenic mothers. The more severe phenotypes of these animals indicates that the loss of *cmtr-1* function impacts other tissues beyond the germline.

In contrast to loss of *cmtr-1, cmtr-2* loss-of-function homozygotes are superficially wild type [6]. To investigate whether loss of *cmtr-2* function influenced the *cmtr-1* loss-of-function phenotype, we examined the phenotype of *cmtr-1; cmtr-2* double mutants. This analysis showed that loss of *cmtr-2* enhanced the phenotype of *cmtr-1* single mutants (Fig. 1H). Thus, CMTR-2 contributes to the role played by CMTR-1 in agreement with results that show *Drosophila* and human CMTr2 can 2′-O-ribose methylate the first nucleotide although with a lower efficiency than CMTr1 [6].

The failure of oocyte differentiation in *cmtr-1* mutant homozygotes suggested that CMTR-1 function is required for some component(s) of the sperm-oocyte switch. To determine when CMTR-1 protein is required during larval development for oocyte cell fate, we generated an auxin-inducible degeneration (AID) allele of *cmtr-1*, which expresses endogenous CMTR-1 tagged with the auxin-inducible degron (AID) peptide and monomeric NeonGreen (mNG^AID::CMTr1). This strain (PE1176) also expresses the *Arabidopsis thaliana* TIR1(F79G) transgene necessary for auxin-dependent protein depletion [23]. Growing this strain in the presence of 5-Ph-IAA showed rapid depletion of mNG^AID::CMTr1 (Supplementary Fig. S1), and such worms were 100% sterile and showed reduced body size compared to untreated controls (Fig. 1I).

Using this strain, we performed a series of experiments designed to deplete mNG^AID::CMTR-1 at specific times during postembryonic development to determine when CMTR-1 function is required for germline cell fate. We initiated synchronous cultures of 50 starved, first-stage larvae (L1s) on control plates and transferred them to 5-Ph-IAA plates at defined times (Fig. 1J). We also performed the inverse experiment, culturing L1s on 5-Ph-IAA plates and transferring them to control plates at the same time points. This ‘auxin-shift’ experiment (named by analogy to temperature-shift experiments performed with temperature-sensitive mutants) revealed that 5-Ph-IAA depletion of mNG^AID::CMTR-1 resulted in sterility only during a critical time period between ~24 and 40 h following initiation of starved L1 larval development. The critical period during which CMTR-1, and thus cOMe, is required corresponds roughly to the L3 and L4 larval stages. Given that the sperm-oocyte switch occurs during the L4 stage [55], the time-window during which CMTR-1 is required suggests that this is when transcripts involved in germline sex determination need to be modified by cOMe for normal germline cell fate.

### The *C. elegans* CMTR proteins show distinct expression patterns and protein–protein interactions

**Figure 2.**
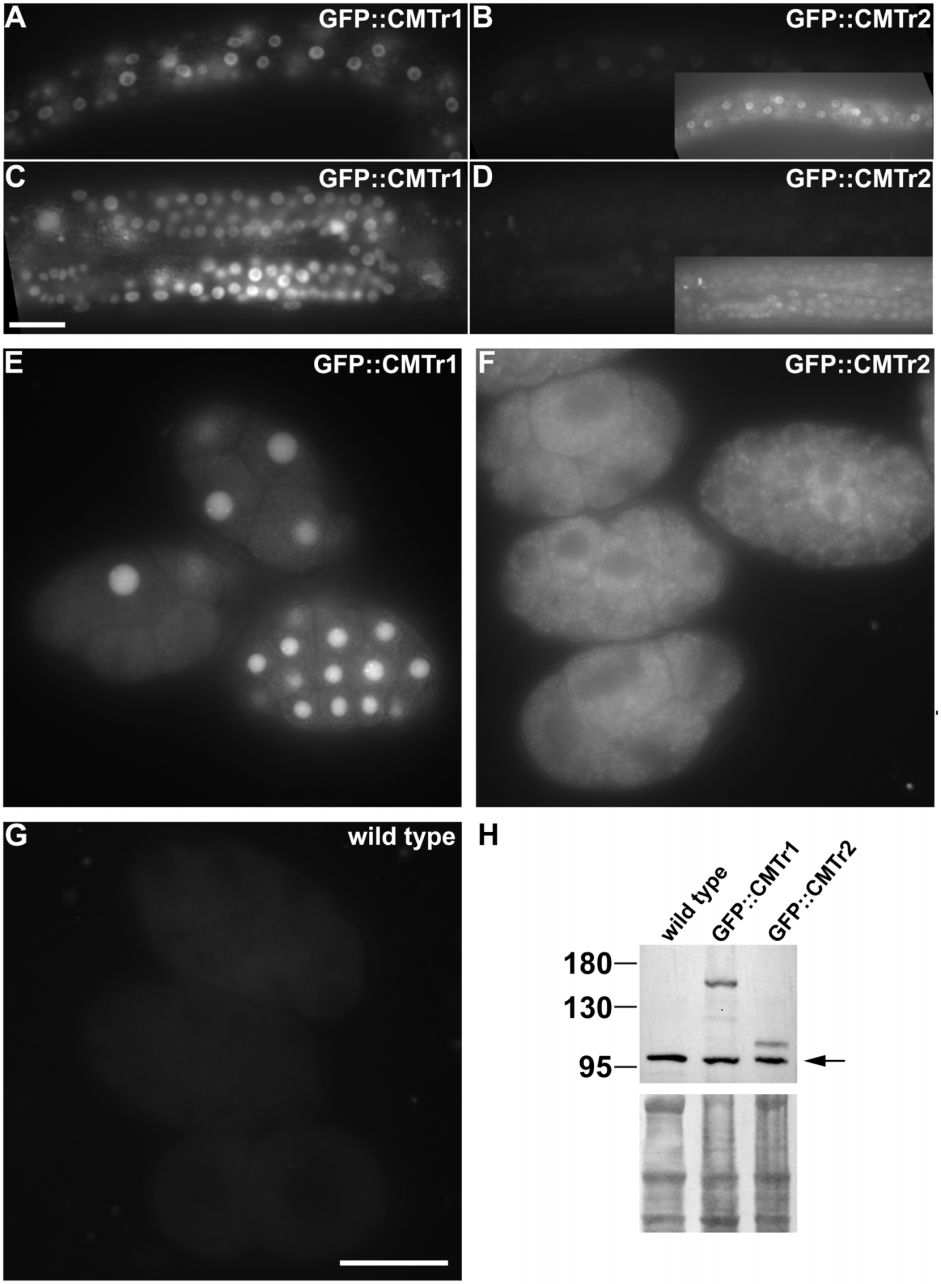
CMTR-1 is constitutively nuclear but CMTR-2 subcellular localization is developmentally regulated. Representative images showing the respective expression of GFP tagged CMTR-1 (A, C, E) or CMTR-2 (B, D, F) in L2 larval stage epidermis (**A, B**), L4 germlines (**C, D**), and early embryos (**E, F**). Wild-type embryos (**G**) show the level of embryonic background autofluorescence. Images in panels A–D and E–G were taken at the same camera exposure settings. Insets in panels B and D are exposure-enhanced. Scale bars indicate 20 µm. (**H**) Western blot of GFP-tagged CMTR-1 and CMTR-2 in embryonic extracts probed with anti-GFP polyclonal antibody. Specific signals are absent from wild-type embryos. Arrow indicates non-specific primary anti-GFP antibody immunoreactivity. Amido black stained immunoblot indicates loading of protein in each lane.

Studies in cultured mammalian cells and *Drosophila* indicated that the two CMTR proteins have conserved, distinct subcellular distributions: in both cases, CMTR1 is primarily nuclear, whereas CMTR2 is both nuclear and cytoplasmic [18, 57, 58]. Using strains that express GFP-tagged alleles of the endogenous proteins, we found that *C. elegans* CMTR proteins are nuclear throughout postembryonic development (Fig. 2A–D), being expressed in most, if not all, cells, though CMTR-2 does not appear to be as strongly expressed in the nucleus as CMTR-1. While the overall levels of the two proteins are similar in embryos, as judged by Western blot (Fig. 2H), the intracellular localization of the two proteins is strikingly distinct: CMTR-1 is predominantly nuclear (Fig. 2E), in contrast to CMTR-2, which is strongly cytoplasmic (Fig. 2F, compare to Fig. 2G).

**Figure 3.**
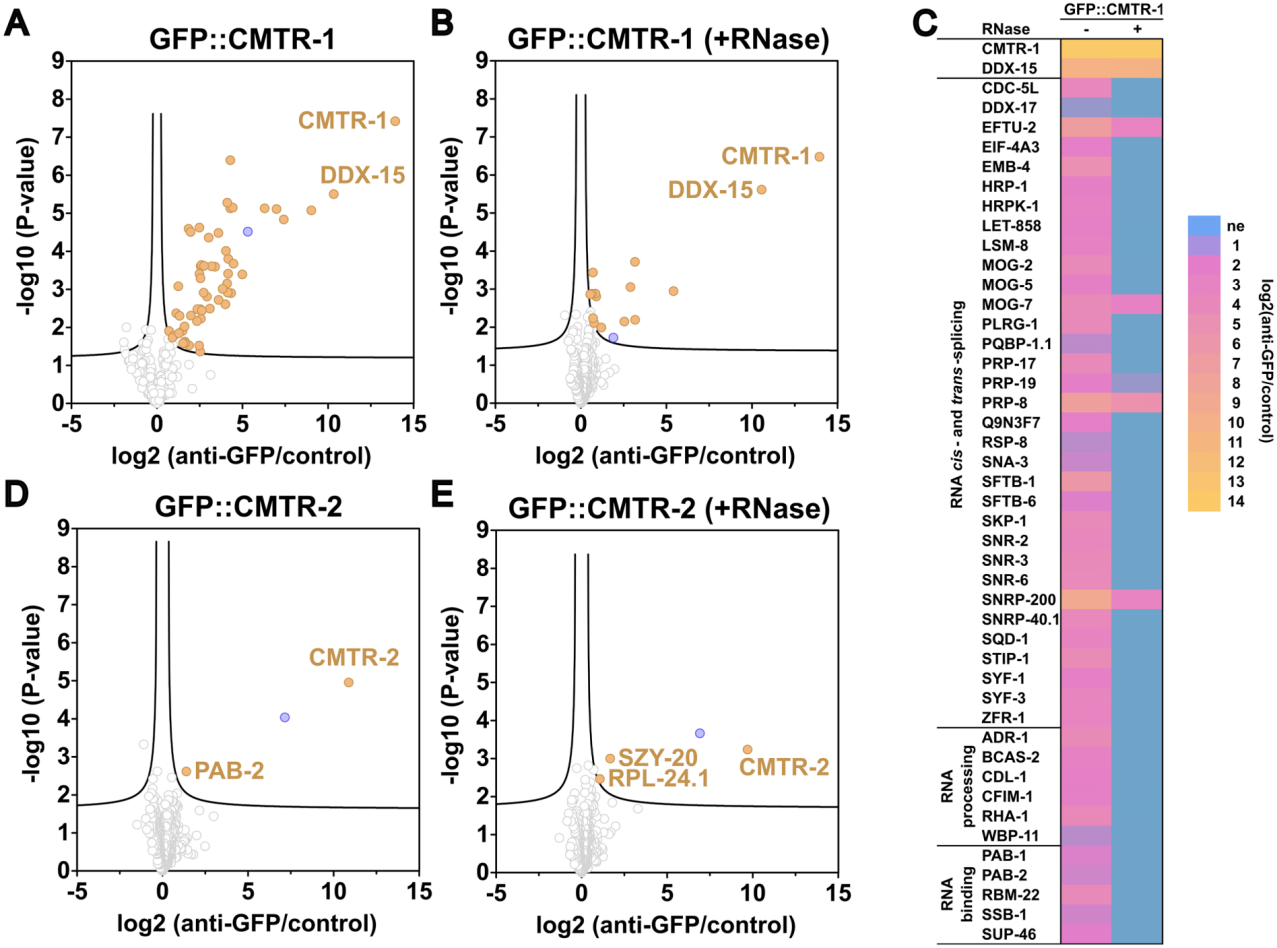
CMTR-1 and CMTR-2 show distinct protein–protein interaction partners. Proteins enriched by co-immunoprecipitation with GFP::CMTR-1 (A, B, C) and GFP::CMTR-2 (D, E) from PE1001 and PE991 embryonic extracts treated without (**A, D**) and with RNase (**B, E**). Immunoprecipitations were performed in triplicate or quadruplicate using anti-GFP nanobodies coupled to agarose beads or control agarose beads. Immunoprecipitated proteins were identified by label-free quantification as described [27]. Graphs show enrichment in anti-GFP nanobody immunoprecipitations (anti-GFP) compared to bead only controls (control) and the false discovery rate (*y*-axis). Proteins that were significantly enriched are shown in gold. The significance cutoff curve is drawn in black (false discovery rate ≤ 0.05 and S0 of 0.1). TBH-1 (blue), a dopamine β-hydroxylase, is found in all immunoprecipitations, but our previous work suggests that it non-specifically associates with GFP-tagged proteins [27, 31]. (**C**) Heatmap summarizing the enrichment of spliceosome components, RNA processing factors, and RNA-binding proteins in immunoprecipitation of GFP::CMTR-1. Proteins were grouped using the GO annotation retrieved from UniprotKB [59–61]. ne: not enriched. For complete lists of identified proteins see Supplementary Table S2.

To investigate the functional significance of the distinct localization of the proteins during embryogenesis further, we performed immunoprecipitation analyses from embryonic lysates to assess their respective protein–protein interactions. This analysis revealed that the two proteins have non-overlapping protein partners: CMTR-1 showed a rich set of protein–protein interactions (Fig. 3A–C), while CMTR-2 displayed a paucity of interactors (Fig. 3D and E), none of them matching those we found for CMTR-1. The lack of overlap in protein interaction partners is consistent with the low levels of CMTR-2 in the nucleus (Fig. 2E and F) in embryos, but this analysis fails to shed light on the functional significance of the cytoplasmic enrichment of CMTR-2.

*C. elegans* CMTR-1 showed broadly the same protein–protein interactions with a suite of pre-mRNA processing factors that were observed for mammalian cells [62], with components of the spliceosome, heterogeneous nuclear ribonucleoproteins, and ribosomal proteins being prominent interaction partners (Fig. 3A–C). We also saw an interaction with SNA-3, a component of the spliced leader *trans*-splicing machinery [31] that has previously been shown to associate with nascent RNA polymerase II transcripts [27].

Since many of the interactions were abolished by RNase treatment, this likely reflects the predicted activity of CMTR-1 in co-transcriptionally binding to nascent transcripts, which has also been shown for the spliceosome. However, there are notable interactions that were preserved in RNase treated samples, including the previously well-defined CMTr1-interacting protein, DHX15 (DDX-15 in *C. elegans*) [63, 64], and some core components of the spliceosomal catalytic complex, notably MOG-7, PRP-8, EFTU-2, and SNRP-200. We suspect that these interactions may be due to interactions between DDX-15 and the spliceosome, as observed in human cells [64].

### Loss of cOMe is the cause of the *cmtr-1* loss-of-function phenotype

**Figure 4.**
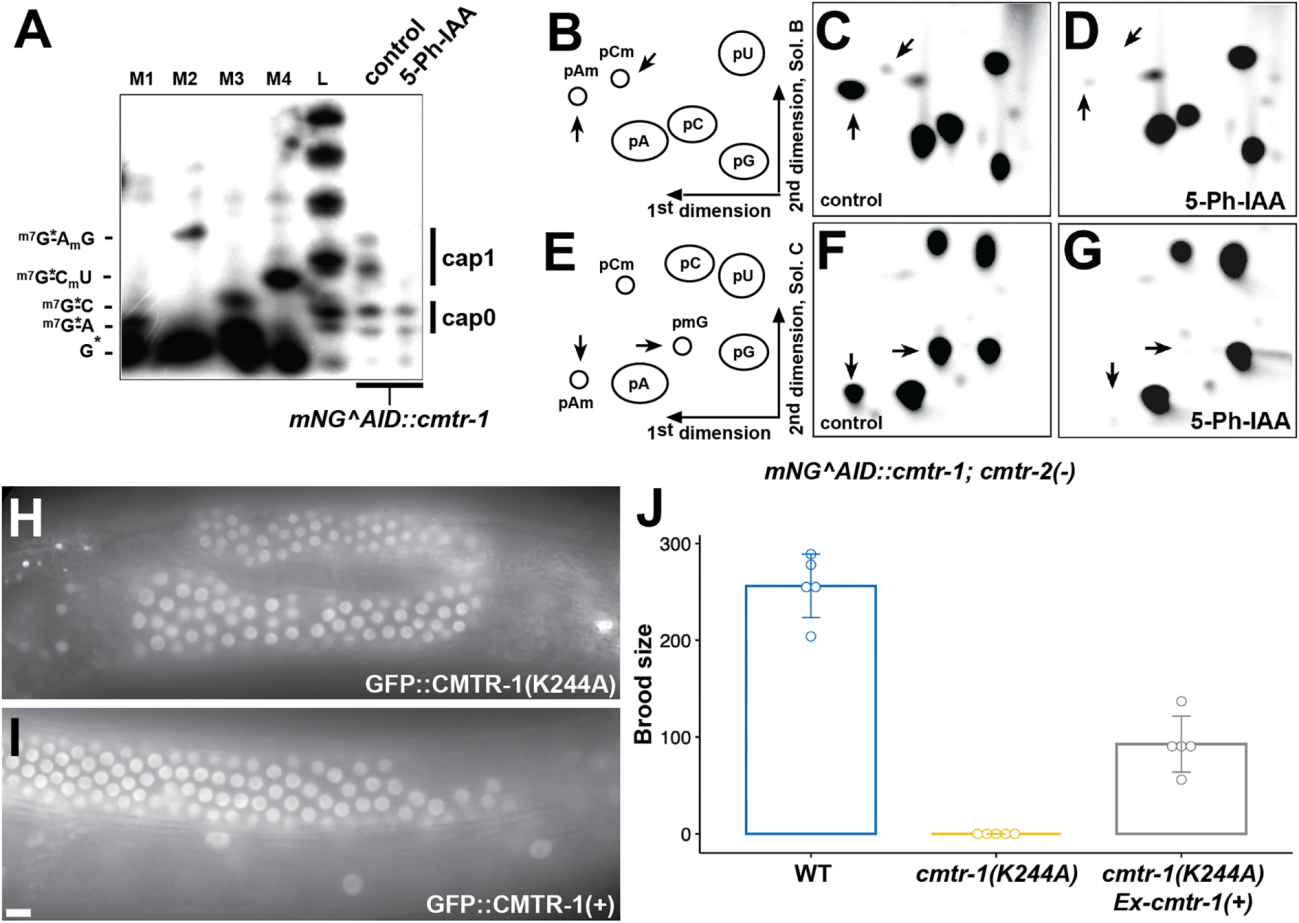
CMTR-1 is required for cOMe in *C. elegans*. Analysis of cap-adjacent 2′-*O*-ribose RNA methylation with (control) and without (5-Ph-IAA) mNG^AID::CMTR-1. (**A**) Recapping assay to detect mRNA using ^32^P-αGTP. 5′ cap structures were separated on a 22% denaturing polyacrylamide gels after digestion with RNase I (‘control’ and ‘5-Ph-IAA’ lanes). Markers (M1–4) are RNase I digested ^32^P-αGTP capped oligonucleotides. 2′-*O*-ribose methylation (*N*_m_) was added using vaccinia CMTr. Sequences of markers are shown on the left. (**B**–**G**) Two dimensional thin-layer chromatograms (2D-TLCs) showing modifications of the cap-adjacent nucleotides prepared from total RNA isolated from worms homozygous for the *mNG^AID::cmtr-1* and *cmtr-2(-)* alleles, grown on control (C, F) or 5-Ph-IAA plates (D, G). (B, E) Schematic diagrams of a 2D-TLC, depicting the positions of unmodified and 2′-*O*-ribose methylated nucleotides. One set of chromatograms (C, D) was run in solvent B for the second dimension, another set (F, G) was run in solvent C. 2′-*O*-ribose methylated nucleotides are indicated by arrows. (**H**) Representative germline GFP fluorescence of an animal expressing GFP-tagged CMTR-1(K244A), compared to GFP-tagged wild-type CMTR-1 protein (**I**). (**J**) Brood counts of wild-type (data from Fig. 1F) and animals homozygous for the K244A mutation, in the absence and presence of the wild-type *cmtr-1* cDNA expressed under the control of the *rps-0* promoter (*Ex-cmtr-1*). Counts were performed on five individual animals per genotype.

The phenotypic impact of loss of *cmtr-1* compared to *cmtr-2* suggested that CMTR-1 function might be more important for cOMe modification of transcripts than CMTR-2. This was supported by our previous analysis, which showed that loss of *cmtr-2* did not detectably affect cOMe levels [6]. To investigate the dependence of cOMe on CMTR-1, we isolated RNA from mNG^AID::CMTR-1 depleted and control fourth-stage larvae. We then subjected this RNA to a sensitive assay based on recapping mRNA with ^32^P-alpha-GTP, followed by RNase I digestion to detect cOMe in small amounts of mRNA [6, 18, 65]. This assay allows us to detect 2′-*O*-ribose methylated transcripts based on that nucleotides with this modification are protected from RNase I digestion. We found that cOMe-modified RNA (cap1) was almost completely abolished (Fig. 4A), demonstrating that CMTR-1 is the main methyltransferase responsible for cOMe modification in *C. elegans*, as it is in *Drosophila*. Note that using this assay, we were not able to detect cap2 modifications, consistent with a previous study that cap2 is largely absent from *C. elegans* [66].

Since CMTR-1 is the key enzyme involved in depositing cOMe, we wanted to determine which nucleotides receive this modification. We subjected RNA isolated from mNG^AID::CMTR1 depleted and control animals to decapping, dephosphorylation, and ^32^P-labelling, before digesting the resultant RNA with nuclease P1 to release individual nucleotides. These were then resolved by 2-dimensional thin-layer chromatography (Fig. 4B–G). This method cannot distinguish between cap1 and cap2 modifications, so to avoid ambiguity caused by possible residual methyltransferase activity arising from CMTR-2 activity, we performed these experiments in a *cmtr-2* null mutant background (using the *tm4453* allele).

In *C. elegans*, most RNA polymerase II transcripts undergo spliced leader *trans*-splicing [67], which replaces the nascent 5′ UTR with short, specialized 22-nucleotide ‘spliced leader’ RNA [68]. There are 20 distinct spliced leaders, but all begin with a guanosine dinucleotide [69]. Since the precursor spliced leader RNAs are RNA polymerase II transcripts, they are predicted to be substrates for CMTR-1, and thus the first nucleotide of most transcripts will be 2′-*O*-ribose methylated guanine. As shown by separation of the second dimension in solvent C, the two major signals that correspond to 2′-*O*-ribose methylated adenine and guanine nucleotides (the signals overlap when the second dimension is run using solvent B) are almost completely abolished by depletion of AID::CMTR-1. While these experiments cannot definitively show that spliced leader RNAs receive cOMe modifications, the results are consistent with this possibility.

Previous studies in mammals have shown that around 50% of CMTR1 is in a complex with DHX15, and that this interaction represses its methyltransferase activity [63, 64]. CMTR1 also influences RNA polymerase II recruitment to transcription start sites and the transcription of histone and ribosomal protein genes [16]. We thus wanted to confirm that the *cmtr-1* loss-of-function phenotype was due solely to loss of cOMe; in other words that it was dependent on CMTR-1 methyltransferase activity.

To address this, we engineered a missense mutation, K244A, into the endogenous *cmtr-1* gene to generate a mutant that expresses a catalytically dead enzyme. The orthologous mutation in human CMTR1 (K239A) has previously been show to abolish methyltransferase function [70, 71], and the lack of function of this mutation in *C. elegans* has been assessed in transgene rescue experiments [19]. We carried out site-directed mutagenesis of the *cmtr-1* allele that expresses CMTR-1 with a N-terminal GFP tag (Fig. 2), allowing us to demonstrate that the K244A mutation did not affect the expression and localization of CMTR-1 (Fig. 4H and I). All worms homozygous for the K244A (*fe175*) allele showed the same recessive sterile and growth-defective phenotypes displayed by the *syb3613* mutation, and these phenotypes were complemented by the *rps-0::cmtr-1(+)* transgene (Fig. 4J). Since these worms specifically lack the methyltransferase activity of CMTR-1, this indicates that loss of cOMe is the cause of the germline phenotypes.

### Loss of CMTR-1 does not affect spliced leader *trans*-splicing

The abundance of 2′-*O*-ribose methylated guanine nucleotides is consistent with cap-adjacent methylation of the spliced leader RNA (Fig. 4E–G). This observation, coupled with the fact that CMTR-1 and SNA-3, an essential component of the SL1 *trans*-splicing machinery [27, 31], showed RNA-dependent interactions (Fig. 3C), led us to investigate the functional impact of cOMe on *trans*-splicing.

**Figure 5.**
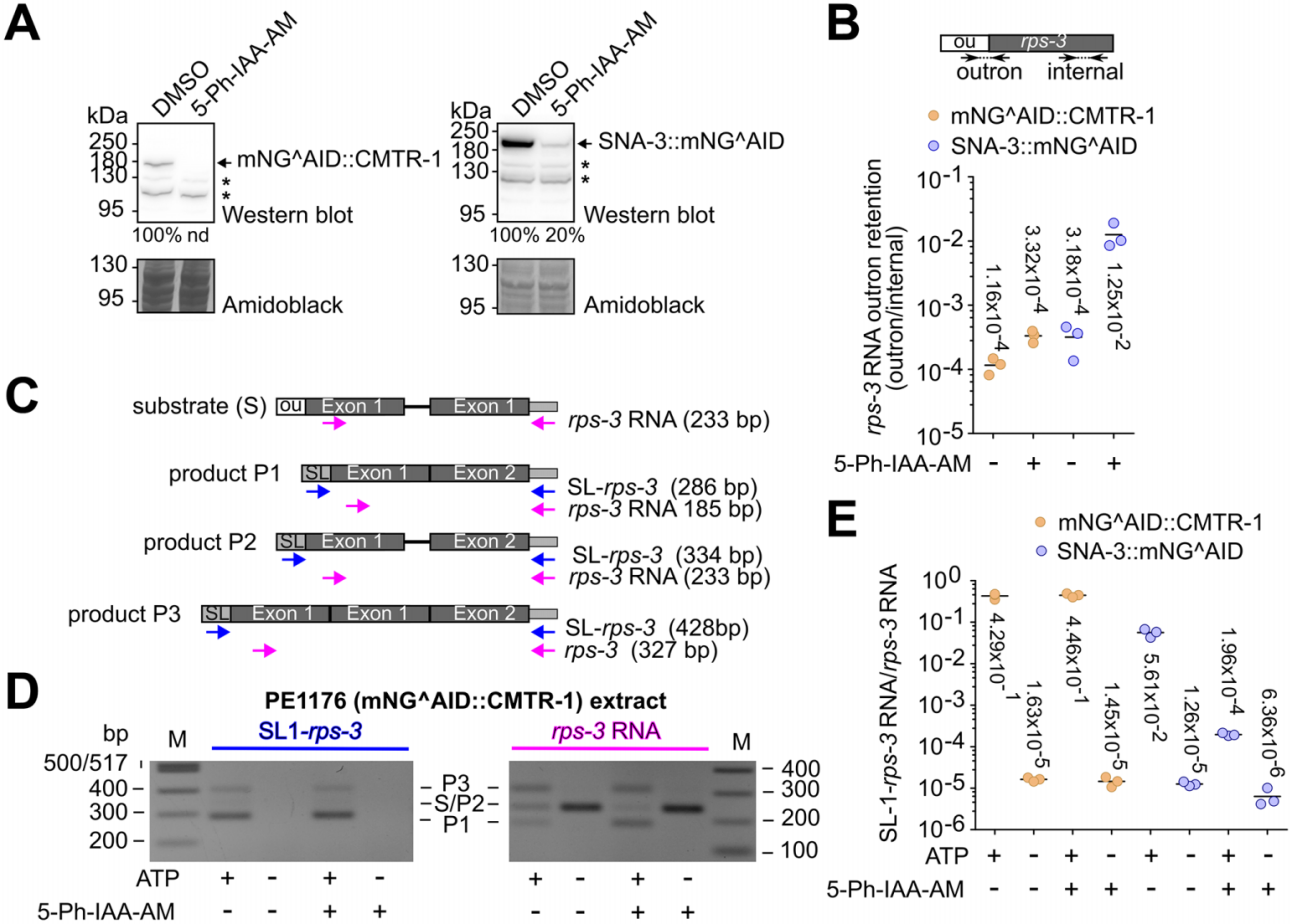
CMTR-1 is not required for spliced leader *trans*-splicing. (**A**) Western blots showing levels of endogenous mNG^AID::CMTR-1 and SNA-3::mNG^AID proteins in embryonic extracts prepared from PE1176 and PE1220 animals, respectively. Embryos were control-treated with DMSO or with the auxin analogue 5-Ph-IAA-AM for 4 h prior to extract preparation. Proteins were analysed by Western blotting and detected by anti- mNeonGreen antibodies. Total protein visualized using amido black staining was used to standardize the levels of mNeonGreen-tagged proteins. The level of SNA-3::mNG^AID protein in 5-Ph-IAA-AM treated extract was expressed as percentage of that detected in the DMSO-treated extract. The mNG^AID::CMTR-1 was not detectable in 5-Ph-IAA-AM treated extracts (nd). Non-specific bands are indicated with a star symbol. (**B**) Quantitative RT-PCR measurement of outron retention of endogenous *rps-3* transcripts. The diagram shows the location of primers that detect outron and internal regions of *rps-3* RNA. RNA was isolated from embryonic extracts, reverse transcribed, and analysed by quantitative PCR as described previously [35]. The graph shows the ratio of endogenous *rps-3* outron to internal amplicon levels in PE1176 (gold) and PE1220 (blue) extracts treated with 5-Ph-IAA-AM (+) or control treated with DMSO (−) determined using the ∆*C*_T_ method [36]. Measurements are technical replicates and their means (horizontal line, mean values are shown). The analysis is representative of two independent measurements. (**C**) Substrate and products of *in vitro* SL *trans*-splicing. Diagrammatic representation of the synthetic *rps-3* transcript (substrate S) with outron (ou) at the 5′ end and the products P1, P2, and P3. The locations of primer pairs SL1-*rps-3* and *rps-3* RNA used for detection by one-step qPCR and expected amplicon size are indicated. (**D**) Analysis of *in vitro* SL *trans*-splicing by agarose gel electrophoresis. Synthetic *rps-3* RNA was incubated with embryonic extracts from PE1176 embryos treated with 5-Ph-IAA-AM or control-treated with DMSO (± 5-Ph-IAA-AM) as described. ATP and ATP regeneration system were included where indicated (± ATP). Products were amplified by one-step PCR, analysed by 1.8% agarose gel electrophoresis and visualized by staining with ethidium bromide. (**E**) Analysis of *in vitro* SL *trans*-splicing by quantitative RT-PCR. Synthetic *rps-3* RNA was incubated with embryonic extracts from PE1176 (gold) or from PE1220 embryos (blue) treated with 5-Ph-IAA-AM or control-treated with DMSO (± 5-Ph-IAA-AM) as described. ATP and ATP regeneration system were included where indicated (± ATP). Products were quantified by quantitative one-step RT-PCR as described. The graph shows the ratio of *trans*-spliced SL1-*rps-3* products standardized with respect to total synthetic *rps-3* RNA using the ∆C_T_ method [36]. The *in vitro* processing reactions were done in triplicates. Shown are the results for each reaction and the mean (horizontal line, values are indicated).

We depleted mNG^AID::CMTR-1 in embryos by treating them with 5-Ph-IAA-AM, which is able to penetrate the eggshell [72], and prepared *in vitro* splicing extracts as described previously [37]. These were compared to extracts prepared from untreated embryos and to PE1220 embryos depleted for SNA-3::AID^mNG, which, based on SNA-3’s essential role, is predicted to impair spliced leader *trans*-splicing [31]. 5-Ph-IAA-AM treatment reduced mNG^AID::CMTR-1 and SNA-3::mNG^AID steady-state levels to undetectable levels and by 80%, respectively (Fig. 5A).

We analysed the effect of protein depletion on spliced leader *trans*-splicing in embryonic extracts using two assays: (i) the steady state spliced leader *trans*-splicing levels of endogenous *rps-3* transcripts; and (ii) the *trans*-splicing by embryonic extracts of an exogenous, synthetic *rps-3* transcript prepared by *in vitro* transcription. In the first assay, depletion of SNA-3 led to a 40.6-fold increase in non-*trans*-spliced, outron-containing endogenous *rps-3* RNAs, while the depletion of CMTR-1 had only a minor effect, leading to a 2.75-fold increase of non-*trans*-spliced *rps-3* RNA (Fig. 5B).

SL1 *trans*-splicing of synthetic *rps-3* transcripts was analysed by PCR as previously described [37]. The synthetic *rps-3* RNA used as substrate contains plasmid sequence at the 3′ end, allowing the use of primers that distinguish the synthetic *rps-3* RNA from endogenous *rps-3* RNA (Fig. 5C). Products P1, P2, and P3 (Fig. 5C and D) were identified by sequence analysis and are detected by the SL-*rps-3* primers that amplify SL *trans*-spliced synthetic RNA and the *rps-3* RNA control primers that detect all forms of synthetic *rps-3* RNA. Importantly, SL-*rps-3* primer products were only detected in reactions containing ATP necessary for spliced leader *trans*-splicing to occur (Fig. 5D). Analysis by qPCR shows that depletion of CMTR-1 has a minor effect on *in vitro trans*-splicing of the exogenously added synthetic *rps-3* RNA, with near-identical amounts of product detected in the presence of ATP, independent of whether CMTR-1 was depleted or not (Fig. 5D). In contrast, the depletion of SNA-3 led to a 280-fold reduction of detection of *trans*-spliced products (Fig. 5E). Thus, both assays failed to detect a clear, pronounced effect of CMTR-1 depletion on spliced leader *trans*-splicing, suggesting that neither cOMe nor CMTR-1 has a significant role in this process.

### Loss of the RNA decapping exonuclease EOL-1 bypasses the need for CMTR-1

To better understand the molecular and cellular significance of the global loss of cOMe, we performed an unbiased genetic screen for mutations that suppress the *cmtr-1* loss-of-function phenotype, reasoning that such mutations might allow us to identify components involved in the cellular response to cOMe modified RNAs.

We mutagenized PE1176 worms with ethyl methanesulfonate (EMS) and grew the F2 generation on NGM plates containing 1 µM 5-Ph-IAA. We then screened for the presence of healthy gravid worms, which would indicate suppression of the *cmtr-1* loss-of-function phenotype. Suppression was confirmed by showing that the worms could be maintained over multiple generations on 5-Ph-IAA containing media. Only one strain was retained per F1 culture, to ensure independence of each suppressor mutation. From combined screens of ~20 000 haploid genomes, we recovered 27 independent mutants that were fertile when grown in the presence of 5-Ph-IAA.

We designed the screen with the expectation that we would recover loss of function mutations in the *TIR1(F79G)* transgene, which restore fertility simply by preventing 5-Ph-IAA-depdenent degradation of mNG^AID ::CMTR-1. Such mutations, which constitute a type of informational suppression [73], could be distinguished from *bona fide cmtr-1* loss-of-function suppressors by the fact that they express mNG^AID::CMTR-1 in the presence of 5-Ph-IAA (Supplementary Fig. S2). Using this secondary screen, we found that of the 27 mutants, 21 showed nuclear mNeonGreen fluorescence despite being grown on 5-Ph-IAA. We sequenced the TIR1 coding region from six of the putative TIR1 loss-of-function mutants and confirmed that all were missense mutations (Supplementary Fig. S2).

**Figure 6.**
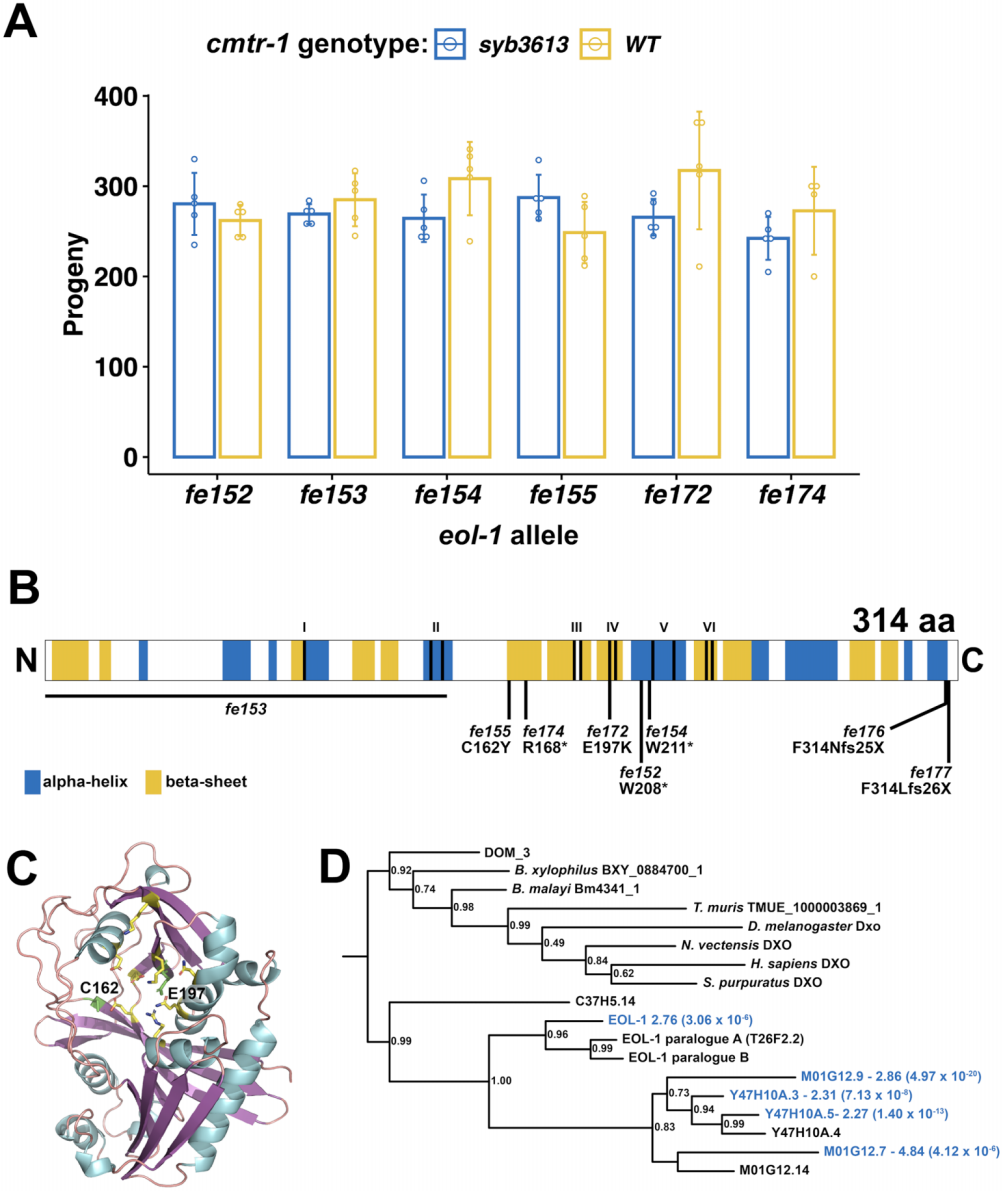
Loss of function mutations in *eol-1* suppress the loss of *cmtr-1* mutant phenotype. (**A**) Brood counts for the six *eol-1* alleles identified as suppressors of *cmtr-1* loss-of-function in *cmtr-1(syb3613)* and *cmtr-1(+)* backgrounds. Histograms show means and error bars show standard deviations for counts derived from five adults for each genotype. (**B**) Schematic of EOL-1 showing the location of the suppressor mutations and the two mutations generated by Cas9/CRISPR (*fe176* and *fe177*). Shading indicates secondary structure assignments based on the AlphaFold prediction (A5JYX9). Roman numerals indicate highly conserved motifs necessary for decapping and exonuclease activity [74]. (**C**) AlphaFold prediction for EOL-1 structure, showing location of residues affected by missense suppressor mutations (green). Active site residues other than E197 are in yellow. (**D**) Phylogram showing the relationship between EOL-1 and other DXO family members. Blue text indicates proteins with transcripts that are significantly enriched in mNG^AID::CMTR-1 depleted animals (Fig. 7). Fold enrichment is given, along with adjusted *P* values in brackets. Node statistical supports are approximate Bayes branch supports (aBayes).

The six mutants that lacked mNeonGreen fluorescence, but were nonetheless fertile, were thus putative suppressors that bypassed the requirement for CMTR-1. We confirmed that they also suppressed the *cmtr-1(syb3613)* null allele: all six mutations suppressed the sterile phenotype and growth defects conferred by the *syb3613* allele confirming that they are epistatic to *cmtr-1* loss-of-function (Fig. 6A).

To identify the gene(s) affected by these six mutations, we used a whole-genome sequencing (WGS) mapping strategy based on the sibling-subtraction approach described previously [49]. We mapped two of the suppressor mutants, *fe152* and *fe154*, using this approach (Supplementary Fig. S3). The sibling-subtraction WGS mapping approach relies on the identification of variants that are present in more than 90% of reads derived from the pooled broods of F2 suppressor animals, but absent, or at a low frequency in the reads derived from their non-suppressor sibling broods. In practice, we found that focussing only on variants found in 100% of suppressor pool reads was sufficient to narrow down the location of the suppressor mutations to a region of chromosome V (Supplementary Table S3). SnpEff and manual curation of these variants revealed predicted damaging variants in a single gene, *eol-1*, allowing us to assign *fe152* and *fe154* as premature stop codon mutations, W208Opal and W211Amber, respectively (Fig. 6B).

Sanger sequencing of the *eol-1* locus in the other four suppressor mutants revealed that each was a predicted damaging allele of *eol-1* (Fig. 6B). A third nonsense mutation is created by *fe174*, R168Opal, and two mutations, *fe155* and *fe172*, are missense mutations, C162Y and E197K, respectively. The *fe153* allele was found to be a 3649 bp deletion that removes 3133 bp upstream of the start codon as well as half of the *eol-1* coding region. The deletion is accompanied by insertion of 349 bp of sequence, part of which appears to be repetitive DNA found on multiple *C. elegans* chromosomes.

Having identified the genetic location of the suppressor mutations, we crossed them into a *cmtr-1* wild-type background, which revealed that they had wild-type brood sizes (Fig. 6A). Thus, loss of *eol-1* function is compatible with wild-type fertility. We also did not detect any obvious phenotypes that would indicate defects in embryonic or postembryonic development.

Although the recovery of six independent function altering mutations in the same gene is strong evidence that loss of *eol-1* function suppresses the requirement for CMTR-1 and thus cOMe, we wanted to formally confirm this. We aimed to delete the *eol-1* gene, using Cas9/CRISPR to generate double-strand breaks at either end of the gene. However, we found that the guide RNA targeted to the 5′-end of the coding region was ineffective, so we were not able to identify gene deletions. Nonetheless, we identified two alleles, *fe176* and *fe177*, that created frameshifts just upstream of the stop codon, both of which would be predicted to result in peptide extensions that derive from translation into the 3′ UTR (Fig. 6B). Proteins with such C-terminal extensions are subject to ‘readthrough mitigation’ mechanisms that lead to downregulation of the affected protein [75–77]. Consistent with this, both alleles suppress the sterile phenotype caused by depletion of CMTR-1.

The fact that seven independently isolated *eol-1* mutations (including two that were targeted specifically to this gene) suppress the *cmtr-1* loss-of-function phenotype demonstrates that loss of EOL-1 bypasses the requirement for *cmtr-1* function. Thus, restoring *eol-1* function should bring about the growth and germline defects we observed in the original *cmtr-1* mutants. To test this, we reintroduced a wild-type *eol-1* transgene (*eol-1(+)*) into PE1234, which allows the 5-Ph-IAA inducible depletion of mNG^AID::CMTR-1 in an *eol-1(fe153*) mutant background. We reasoned that if the suppression of the *cmtr-1* loss-of-function phenotype was caused specifically by absence of EOL-1 function, then in the presence of the *eol-1(+)* transgene, depletion of mNG^AID::CMTR-1 would result in restoration of growth defects and a sterile phenotype. Worms lacking the transgene would, however, show normal growth and fertility. Finally, both transgenic and non-transgenic worms grown in the absence of 5-Ph-IAA would express mNG^AID::CMTR-1, and thus both show wild-type development and fertility.

We tested three independent transgenic lines (PE1369-71) and allowed broods to hatch in the presence or absence of 5-Ph-IAA. *Caenorhabditis elegans* transgenic lines carry extrachromosomal transgenic arrays that are semi-stable [28], so each line segregated transgenic and non-transgenic broods (recognized by presence or absence of tdTomato fluorescence in the pharynx, respectively). We scored each brood for adult fertility (*n* > 100 transgenic/non-transgenic animals per brood). As predicted, 100% of *mNG^AID::cmtr-1; eol-1(fe153)* mutants carrying the *eol-1(+)* transgene showed severe growth defects, with those reaching adulthood being sterile. These phenotypes were absent in non-transgenic animals grown on 5-Ph-IAA or transgenic and non-transgenic animals grown on control plates. Thus, restoring wild type EOL-1 function to *cmtr-1*(-); *eol-1*(-) double mutants recapitulated the phenotypes caused by loss of *cmtr-1* alone, formal proof that EOL-1 function is the determinant of the phenotypic impact of loss of *cmtr-1* function.

EOL-1 is a member of the DXO/Rai1 family of decapping enzymes [78–81], and some members are also distributive 5′–3′ exonucleases: they can both decap and degrade their target RNAs. The missense mutations, C162Y and E197K, alter residues that are part of the predicted active site of EOL-1 (Fig. 6C). Indeed, E197 is the homologue of mammalian DXO E253, a critical catalytic residue, part of the so-called Motif IV [78, 79]. Thus, EOL-1 loss of function mutations which specifically abolish its decapping/exonuclease activity, completely suppress the sterility caused by loss of CMTR-1, indicating that removing this activity is likely the mechanism which mediates the suppression.

**Figure 7.**
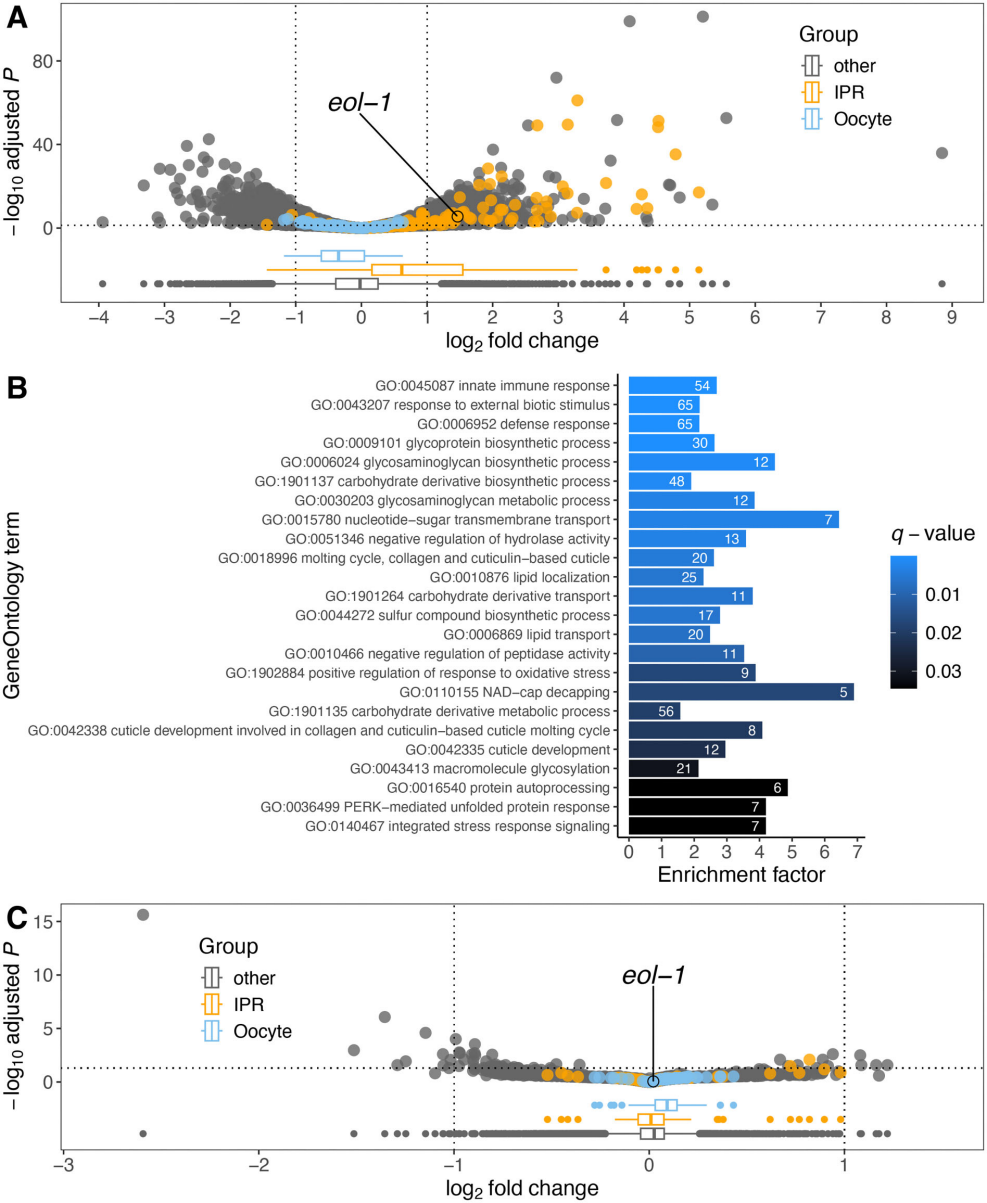
Depletion of CMTR-1 results in EOL-1-dependent upregulation of transcripts associated with the innate immune response and downregulation of transcripts involved in germline sex determination. (**A**) Volcano plot showing differential gene expression in mNG^AID::CMTR-1 animals subject to 5-Ph-IAA-induced depletion compared to controls (three biological replicates). IPR are highlighted in orange. Germline sex determination genes (oocyte) are highlighted in blue. The *eol-1* gene is specifically labelled. The box plots below summarize fold changes among the three gene groups. Welch two-sample *t*-tests for the differences between the means of the IPR and oocyte gene sets and the ‘other’ (non-IPR/germline sex determination genes) were highly significant (*P *< .001). (**B**) GO enrichment analysis showing the significantly overrepresented terms among upregulated genes (log_2_ FC > 0; FDR ≤ 0.05) in animals subject to CMTR-1 depletion compared to controls. GO terms are ranked by *q*-value (most significantly overrepresented terms on top). Numbers of DEGs observed in each term are presented at the edge of each bar. Enrichment factor denotes the observed gene ratio divided by the background gene ratio. (**C**) Volcano plot showing that the IPR and germline sex determination gene expression changes caused by depletion of mNG^AID::CMTR-1 are dependent on EOL-1 function, as they revert to base-line in the absence of EOL-1. Gene expression in *mNG^ AID::cmtr-1; eol-1(fe172[E197K])* double homozygotes subject to 5-Ph-IAA induced depletion compared to those subject to control treatment (six biological replicates). Box plots summarize distribution of the three gene groups shown in panel (A).

Most animals, including many nematodes, have a single DXO homologue (Fig. 6D). In contrast, *C. elegans* has eleven, which are likely the product of multiple gene duplication events. There are two tandem gene clusters on chromosome I (*M01G12.7*- *M01G12.9*-*M01G12.14*; and *Y47H10A.5*-*Y47H10A.4*-*Y47H10A.3*), and one on chromosome V (*eol-1* itself, and two genes upstream of it, designated paralogues A and B). There is also a single phylogenetically distinct homologue, C37H5.14, present at a different location on chromosome V. RNA-Seq analysis showed that only *eol-1* transcripts, along with four of the other ten paralogues are upregulated by depletion of CMTR-1 (see below, Fig. 7 and Supplementary Table S4). It is intriguing that we recovered suppressor mutations only in *eol-1* and none in its paralogues, suggesting that only this gene has sufficient relevant function to respond to cOMe-less RNAs.

### Loss of cOMe leads to *eol-1*-dependent differential transcript levels for genes involved in the intracellular pathogen response and germline sex determination

To investigate the possible impact of loss of cOMe on gene expression, we compared transcript levels for 5-Ph-IAA treated to control-treated mNG^AID::CMTR-1 animals. We cultured synchronized populations of mNG^AID::CMTR-1 larvae and began the 5-Ph-IAA treatment when the animals were at the third larval (L3) stage, corresponding to the developmental stage at which CMTR-1 is critical for the sperm-oocyte switch (Fig. 1J). Both control and 5-Ph-IAA treated animals were harvested for RNA preparation at the early L4 stage.

We performed differential gene expression analysis, comparing RNA-Seq derived from control versus 5-Ph-IAA treated animals. This revealed 1953 genes whose steady state transcript levels were significantly upregulated by mNG^AID::CMTR-1 depletion (FDR ≤ 0.05, log_2_ fold change > 0), and 3398 genes for which transcript levels were significantly reduced by the same treatment (FDR ≤ 0.05, log_2_ fold change < 0) (Supplementary Table S4). Nearly all (96%) of these DEGs were protein-coding genes, which represents a significant enrichment (chi-square test: *P* < .001) compared to the background proportion (69% of assayed genes are protein-coding). Non-coding RNA biotypes made up only 4% of all DEGs and most of these were upregulated, showing the opposite regulation pattern of protein-coding genes (Supplemental Table 4).

Inspection of the 111 genes that were significantly upregulated more than four-fold in mNG^AID::CMTR-1 knock-down worms compared to controls revealed 28 genes, including *fbxa-158, pals-14*, and *pals-6*, which belong to a class of genes that constitute the IPR, a *C. elegans* transcriptional stress response triggered by intracellular pathogens [21]. IPR is induced by viral and microsporidia infections, but also mitochondrial dysfunction [82, 83]. To systematically investigate the possibility that IPR genes were generally upregulated in response to depletion of CMTR-1, we extracted the fold changes of 272 IPR genes that are known to be upregulated more than four-fold in response to expression of Orsay virus RNA-dependent RNA polymerase [82] (Fig. 7A). These IPR genes showed statistically significantly higher fold changes in response to CMTR-1 depletion compared to non-IPR genes (*P* < .001), though the effect size was modest (mean fold change: 0.697) (Fig. 7A). The effect of loss of CMTR-1 on innate immune gene expression was also supported by GO term enrichment analysis (Fig. 7B): the three most significantly overrepresented GO terms among upregulated genes were ‘innate immune response’ (GO:0 045 087, *q*-value = 2.02 × 10^−8^), ‘response to external biotic stimulus’ (GO:0 043 207, *q*-value = 3.92 × 10^−7^), and ‘defense response’ (GO:0 006 952, *q*-value = 4.20 × 10^−7^). Thus, loss of CMTR-1 and cOMe leads to transcriptional upregulation of many IPR genes.

GO term enrichment analysis among downregulated genes in response to loss of CMTR-1 highlighted a broad range of metabolic terms but, intriguingly, also terms related to gametogenesis, including ‘gamete generation’ (GO:0 007 276, *q*-value = 3.59 × 10^−13^) and ‘germ cell development’ (GO:0 007 281, *q*-value = 1.96 × 10^−9^) (Supplementary Table S4). To investigate whether transcripts from genes associated with germline sex determination were downregulated in response to depletion of CMTR-1, we extracted the fold changes of a set of 63 genes involved in germline sex determination (Fig. 7A). We found that this set of genes showed statistically significantly (*P *< .001) lower fold changes than all other genes, though as for the IPR gene response, the effect size was modest (mean fold change: −0.287). Nonetheless, since germline development is known to be highly sensitive to shifts in gene expression [84], this overall trend of downregulation may explain the germline defects that we observed (Fig. 1).

We were unable to establish a clear correlation between transcript level changes and the growth defects that we observed in animals depleted of CMTR-1. It is likely that changes in the expression of a broad range of genes are responsible for this phenotype.

Given that loss of EOL-1 suppresses the mutant phenotype caused by loss of cOMe, we wanted to understand how this related to the gene expression changes we observed in worms lacking CMTR-1. We suspected that loss of EOL-1 function would reverse the changes in germline sex determination genes that we observed in animals depleted for CMTR-1 function. However, we also wanted to determine the role of EOL-1 function on the IPR transcriptional response. To investigate this, we performed depletion of mNG^AID::CMTR-1 in an *eol-1* mutant background, using the *fe172* allele, since this was predicted to specifically affect EOL-1 catalytic activity. This revealed that in the absence of EOL-1 function, there were relatively few genes [50] that showed changes in transcript levels in response to mNG^AID::CMTR-1 depletion (Fig. 7C and Supplementary Table S4). This is consistent with the wild-type phenotype displayed by *cmtr-1; eol-1* double mutants. As predicted, loss of EOL-1 function corrected the reductions in germline sex determination gene expression (Fig. 7C). However, strikingly we also found that the IPR transcriptional response was abolished, indicated that its induction in animals lacking CMTR-1 is dependent on EOL-1 activity. This suggests that it is not the presence of cOMe-less RNAs that directly induce the response, rather it is the global impact on gene expression, caused by EOL-1 acting in response to these RNAs, that induces the IPR.

To further understand the functional significance of the IPR induction and the phenotype due to lack of CMTR-1, we investigated the role of the *C. elegans* RIG-I homologue, DRH-1, on the *cmtr-1* loss-of-function phenotype. DRH-1 is required for induction of the IPR in response to viral infection and mitochondrial dysfunction [81, 82], so we tested whether loss of *drh-1* function could suppress the mutant phenotype of *cmtr-1* null homozygotes. The phenotype of *cmtr-1(syb3613); drh-1(ok3495)* double mutants was indistinguishable to that of *cmtr-1(syb3613)* single mutants (*n* = 495), indicating that DRH-1-dependent induction of the IPR does not significantly contribute to the phenotypic defects caused by loss of CMTR-1 function.

**Figure 8.**
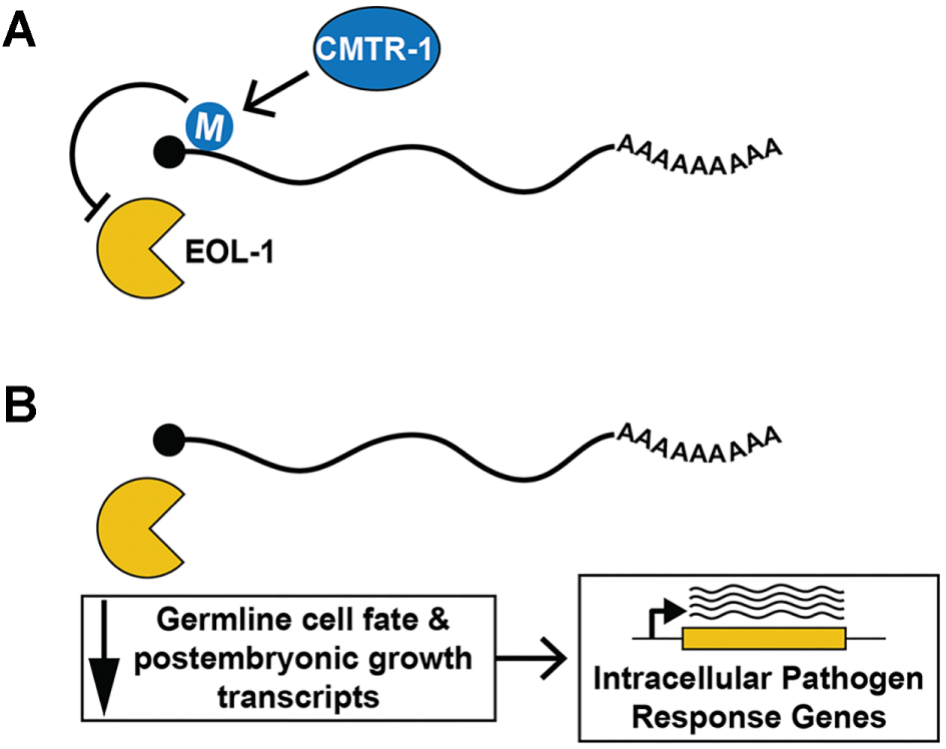
EOL-1 is epistatic to cOMe. (**A**) CMTR-1-mediated deposition of cOMe (M) inhibits EOL-1 activity. (**B**) In the absence of CMTR-1, loss of cOMe potentiates EOL-1 function, leading to reduced steady-state levels of mRNAs that promote wild-type germline cell fate (and likely also postembryonic growth, though these are less well-defined). The upregulation of the IPR is likely a stress response caused by globally altered gene expression as a result of EOL-1 activity.

Taken together, our data demonstrate that loss of CMTR-1 results in accumulation of cOMe-less RNAs that cause profound gene expression changes via a mechanism that requires EOL-1 decapping/exonuclease activity (Fig. 8). Since induction of the IPR is dependent on EOL-1 function, it seems likely that this is a stress-response to widespread gene expression changes, rather than the cOMe-less RNAs directly inducing the response as part of a foreign RNA surveillance response, as is the case in mammals [85].

## Discussion

Insights into the role of cOMe in animal development require studying the effects of loss of the CMTR proteins in the context of whole-organism biology. While work in mice and flies has advanced our understanding of cOMe biology, the fact that these RNA modifications are essential for mammalian embryogenesis but dispensable in flies and some cultured mammalian cells is puzzling. By investigating cOMe function in *C. elegans*, we have obtained a broader evolutionary perspective of the role of these RNA modifications.

We find that CMTR-1 is the principal enzyme involved in adding cOMe to *C. elegans* RNAs, and consistent with this, has higher steady-state nuclear levels compared to CMTR-2. Similarly consistent with these observations, CMTR-1 is essential while CMTR-2 is not. We did not find a function for CMTR-2 beyond augmenting that of CMTR-1, which suggests that CMTR-2 is also able to modify the first nucleotide, a substrate specificity also found for its *Drosophila* and human homologues in modifying canonical AGU starting transcripts [6, 18, 65].

The two nematode CMTR proteins have distinct sub-cellular localizations and interaction partners, suggesting that they are functionally differentiated. Much of this may be due to their differing subcellular localizations. As in mammals and *Drosophila* [18, 57], we see stronger associations of CMTR-2 with the cytoplasm. This is most pronounced during embryogenesis where CMTR-2 is apparently excluded from the nucleus, something not reported in other animal cells. This observation warrants further investigation.

The protein interaction profile of *C. elegans* CMTR-1 is highly similar to its mammalian homologue [62], with both proteins showing prominent interactions with the spliceosome. The functional significance of these observations is unclear but may simply reflect the fact that these proteins assemble on nascent transcripts. In *Drosophila CMTr1* and *2* double knock-outs, no differences in alternative splicing were detected [18], but it is possible that CMTR-1 has a functional role in *cis*-splicing of certain transcripts. However, our experiments do indicate that CMTR-1 is not an essential component of nematode spliced leader *trans*-splicing. Despite the interaction between CMTR-1 and SNA-3, neither of our functional assays indicates that CMTR-1 is required for the activity of the *trans*-spliceosome.

Since cOMe plays an essential physiological role in *C. elegans*, this has allowed us to fully exploit the advantages of this genetically tractable model system to understand the mechanistic basis of the cellular response to cOMe. Our work contributes to the burgeoning field of epitranscriptomics, which involves the study of the role of cap-adjacent methylation events in the regulation of RNA stability. The modification deposited by CMTR-1 seems likely to be constitutive, but another, the m^6^A_m_ modification, is dynamic and reversible, providing a mechanism to regulate mRNA stability [86]. However, perhaps surprisingly, cap-adjacent m^6^A_m_ and the enzymes that add and remove it are absent from *C. elegans* [87]. Thus, our work is the first demonstration in this organism of the importance of cap-adjacent methylation as a mechanism to regulate the physiologically relevant activity of a decapping exonuclease. This confirms and extends a previous *in vitro* study which showed that cOMe reduces the affinity of DXO for RNA and blocks its exonuclease activity [88].

In the light of this data, coupled with the fact that the CMTR-1 loss of function phenotype is apparently completely abrogated by loss of EOL-1 activity, this suggests that the appearance of transcripts lacking cOMe modifications, renders them able to bind to and be degraded by EOL-1. Removal of EOL-1 activity would thus be predicted to return these cOMe-less transcripts to near wild-type levels, which is what we observe for the transcripts associated with germline sex determination when CMTR-1 is depleted in EOL-1(E197K) mutants. Since this particular mutation is critical for the exonuclease activity in yeast DxoI [78], this indicates that loss of EOL-1 exonuclease activity is key to the suppression of both the cellular and molecular phenotypes caused by loss of CMTR-1 function. Future experiments will of course be necessary to confirm EOL-1 *in vivo* decapping and exonuclease activity.

It is perhaps striking that loss of a single enzyme should compensate for the molecular and cellular defects caused by the absence of a key conserved RNA modification. Moreover, since animals lacking both cOMe and EOL-1 together, or EOL-1 alone appear essentially wild-type, at least under laboratory culture, this suggests the apparently paradoxical situation where cOMe modifications exist simply to repress the activity of EOL-1.

Previous studies have proposed that DXO proteins maintain RNA quality control, removing partially capped RNAs that lack cOMe before they enter the cytoplasm [79]. They have also been shown to have a high affinity for NAD-capped RNAs, which are targeted by DXO for rapid degradation [89]. Such roles would be consistent with our data. However, EOL-1 is a prominent component of the IPR, and recent work published while this manuscript was in revision showed that reduced CMTR-1 function renders worms resistant to *P. aeruginosa*, which provides the first evidence of a role for cOMe as part of the innate immune response [20].

EOL-1 was first identified in screens for mutants with enhanced aversive behavioural responses to pathogenic bacteria [80], and it is a notable member of the IPR, induced by Orsay virus, a positive sense, single-stranded RNA virus, and the microsporidian *Nematocida parisii* [90, 91]. It should be noted that despite its name, the IPR is also induced by mitochondrial dysfunction and proteotoxic stress [92–94], though these effects may all be induced by pathogenic infection. Mutations associated with mitochondrial dysfunction also result in elevated *eol-1* expression levels [81, 95], with EOL-1 protein undergoing changes in its intracellular localization, forming cytoplasmic foci in response to mitochondrial defects [81].

Like many individual components of the IPR, a mechanistic role for EOL-1 in this response has yet to be defined. Our work connecting EOL-1 to cOMe suggests a possible function for this protein in the surveillance for RNAs lacking cap-adjacent methylation structures. Thus, mitochondrial dysfunction, defective proteostasis, and other cellular stresses would be interpreted by the cell as possible infection events. EOL-1 as member of an enzyme family that can only target RNAs that lack cOMe modifications would be a useful component of such a response. For instance, Orsay virus are dependent on cOMe-less RNAs for their infection cycle [96]. This may explain the near universal elevation of EOL-1 transcripts in response to infectious agents and internal stresses. Thus, the identification and removal of pathogenic RNAs on the basis of cOMe absence is a plausible function of this enzyme in *C. elegans* that warrants further exploration.

The relationship we have discovered between cOMe and EOL-1 may have broader phylogenetic significance, with DXO proteins having a conserved role in RNA surveillance based on cOMe status. In particular, this may explain the molecular basis of the lethality observed in mammalian embryos lacking either of the two CMTRs [12]; in the light of our work, perhaps loss of cOMe in mouse embryos renders many cellular transcripts targets of mammalian DXO. While *DXO* transcripts are not upregulated by the loss of cOMe [12], unlike those of *eol-1* in *C. elegans*, cellular levels of DXO may still be sufficient to have an impact across the transcriptome. Thus, the embryonic lethality observed in embryos lacking cOMe might be explained by the cumulative downregulation of multiple transcripts. It will be important to determine whether loss of DXO can suppress the embryonic lethality of loss of *Cmtr1* in mice.

Multiple studies in diverse experimental systems have described roles for cOMe at multiple steps in the regulation of gene expression, including transcription, splicing, and translation [13–18]. Our results showing that in *C. elegans* lacking EOL-1, cOMe is largely dispensable, at least in laboratory culture, would seem at odds with these data. However, our results are consistent with work showing that in certain mammalian cell types, the impact of loss of cOMe is relatively minor [12]. Similarly, loss of cOMe in *Drosophila* does not result in profound cellular impacts, with such animals being viable and fertile [18]. The ubiquity of cOMe and the conservation of the two CMTR proteins that catalyse it shows that it must have an essential role across animal phylogeny. Perhaps its original, ancestral role was in RNA-based immune surveillance, which has been preserved in *C. elegans* and mammals (but apparently lost in *Drosophila*), with its functions in gene expression having arisen due cellular adaptation to the presence of cOMe on cellular RNAs. Alternatively, it may have been co-opted into roles in innate immunity through convergent evolution in mammals and nematodes from an ancestral function(s) in the regulation of gene expression. Better understanding of the molecular impacts of cOMe loss in diverse physiologically relevant experimental systems will be needed to distinguish these hypotheses.

## Supplementary Material

gkag355_Supplemental_Files

## Data Availability

The mass spectrometry proteomics data have been deposited to the ProteomeXchange Consortium via the PRIDE [97] partner repository with the dataset identifier PXD058991 (See also Supplementary Table S2). The RNA-Seq data used for the differential gene expression analyses have been deposited in ArrayExpress under accession numbers E-MTAB-14816 (mNG^AID::CMTR-1 depletion, *eol-1* wild-type) and E-MTAB-16687 (mNG^AID::CMTR-1 depletion, *eol-1(fe172)*). Sequence reads that were used in the sibling selection whole genome sequence mapping of *eol-1(fe152)* and *eol-1(fe154)* are available in the NCBI Sequence Read Archive under BioProject ID PRJNA1216994.
