# Supplementary material for: Cap-adjacent 2′-O-ribose methylation of RNA in C. elegans is required for postembryonic growth and germline development in the presence of the decapping exonuclease EOL-1
Source: Nucleic Acids Res. 2026 Apr 30;54(8):gkag355. doi: 10.1093/nar/gkag355 (PMC13129544; doi:10.1093/nar/gkag355)
Supplement: gkag355_Supplemental_Files [file gkag355_supplemental_files.zip › NAR-01820-Z-2025.R2_Supp_Figs.docx]

**SUPPLEMENTARY TABLES AND FIGURES**

**Supplementary Table 1. Strains and oligonucleotides used to generate plasmids and transgenes as outlined in Materials and Methods.** These include oligonucleotides used to: (i) generate plasmids and transgenes for *cmtr-1(syb3613)* and *eol-1(fe172)* homozygote rescue experiments; (ii) generate guide RNA expression plasmids and homology repair templates to make the *GFP::cmtr-1/-2* knock-in alleles; (iii) generate guide RNA expression plasmids and homology repair templates to make the *mNG^AID::cmtr-1* knock-in alleles; (iv) generate guide RNA expression plasmids and homology repair templates to engineer the *cmtr-1* and *eol-1* mutations; (v) detect status of *cmtr-1*, *cmtr-2*, *eol-1*, *TIR1* and *drh-1* alleles; (vi) detect spliced leader *trans*-splicing products.

**Supplementary Table 2. Identification and quantitation of proteins interacting with GFP-tagged CMTR-1 and CMTR-2 proteins.** Worksheets detail the results for each GFP-tagged protein, including the *C. elegans* strain used in each case, and whether or not the samples were incubated with RNase.

**Supplementary Table 3. Differential gene expression analysis of mNG^AID::CMTR-depletion compared to controls in *eol-1* wild-type and *fe172* backgrounds.** Worksheets detail the differential gene expression data for each gene, the Biotype analysis results, and the GO enrichment data for genes that respond to mNG^AID::CMTR-1 depletion.

**Supplementary Table 4.** **Variants found in 100% of suppressor pool reads for *eol-1* alleles *fe152* and *fe154*.** Light blue highlight indicates the same variant found in both *fe152* and *fe154* Sup pools. Yellow highlight indicates the respective predicted *eol-1* damaging variants.

**
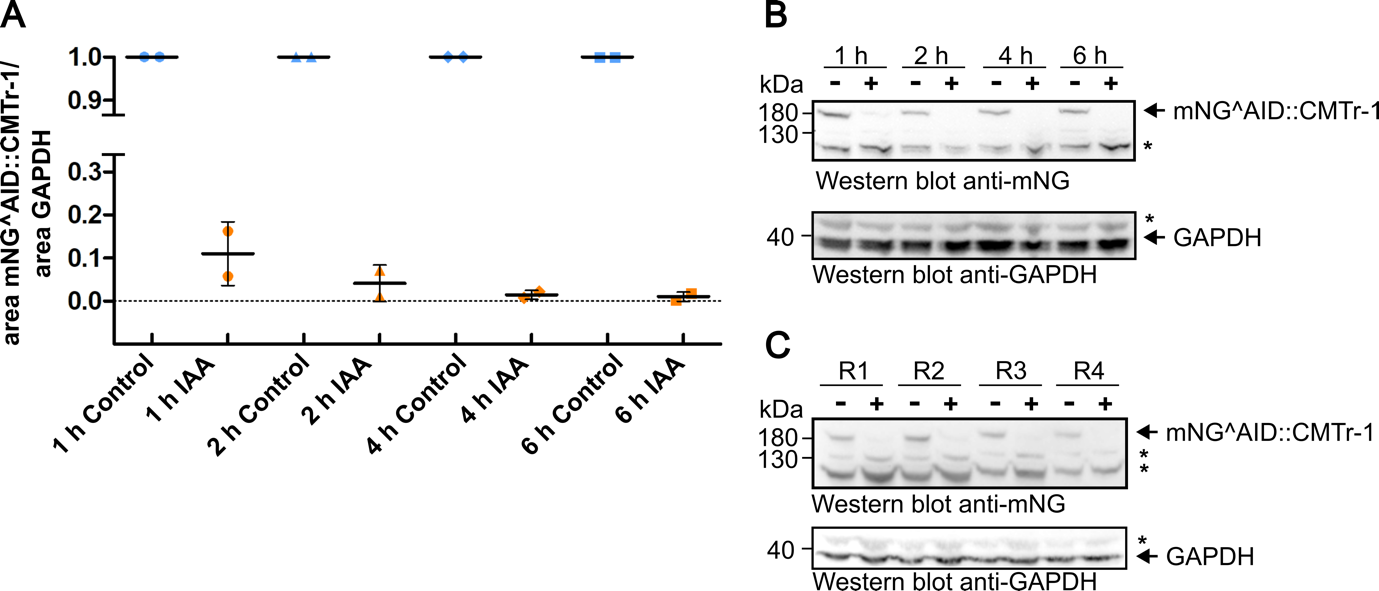
**

**Supplementary Figure 1. 5-Ph-IAA depletion of mNG^AID::CMTR-1.** (A) PE1176 animals were treated with 5-Ph-IAA or control-treated and subjected to Western blotting at the indicated time points. Proteins were detected using anti-NeonGreen and anti-GAPDH antibodies. Protein levels of mNG^AID::CMTR-1 were standardised relative to GADPH, with the controls set to 1. Data represents 2 biological replicates. (B) Representative Western blot for the data shown in A. (C) Western blot of mNG^AID::CMTR-1 depletion of replicates (R1-4) used to generate RNA-Seq data (see Figure 7). In B and C, “+” indicates 5-Ph-IAA treated samples and “-“ control samples, respectively. The “+” and “-“ samples for R1 and R3, and R2- and R4+ were sent for library and Illumina sequencing (Novogene). Designations used R2- = Contr_1; R1+ = IAA_1; R1- = Contr_2; R4+ = IAA_2; R4- = Contr_3; R3+ = IAA_3.


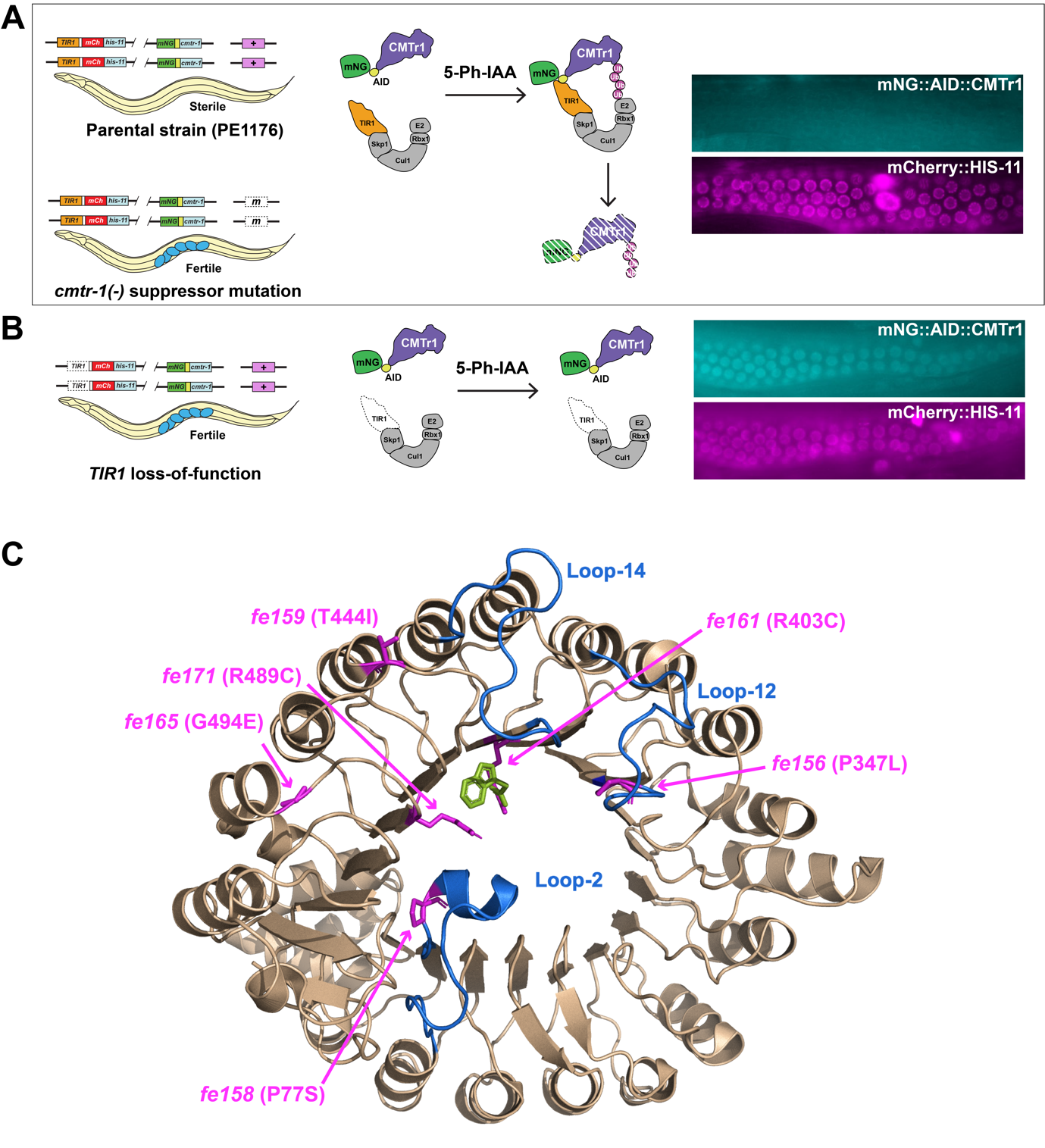


**Supplementary Figure 2. Secondary screen to distinguish suppressors epistatic to *cmtr-1(-)* from *TIR1* loss-of-function mutants.** (A) The starting strain for the suppressor mutagenesis screen (PE1176) is sterile, while strains carrying suppressor mutations (m, unshaded, dashed box) that are epistatic to *cmtr-1(-)* restore fertility (blue ovals indicate eggs inside gravid hermaphrodites). (B) We predicted that TIR1 loss-of-function mutants (unshaded, dashed box) would also restore fertility to *cmtr-1(-)* animals, since these would prevent depletion of mNG^AID::CMTR-1. To distinguish between these two suppressor classes, we conducted a secondary screen based on mNeonGreen fluorescence. This was absent in PE1176 and epistatic suppressor strains (panels show distal gonad arms of adult hermaphrodites; nuclear mCherry::HIS-11 fluorescence was unaffected and serves to locate the germline nuclei), but was present in *TIR1* loss-of-function mutants. (C) Location of selected *TIR1* loss-of-function mutations identified from secondary screening (magenta). View of the TIR1-LRR domain showing the auxin and substrate-binding pocket (<https://doi.org/10.2210/pdb2P1Q/pdb>; (99)). Auxin is shown in green, the three extended loops key for the formation of the pocket are shown in blue.

**
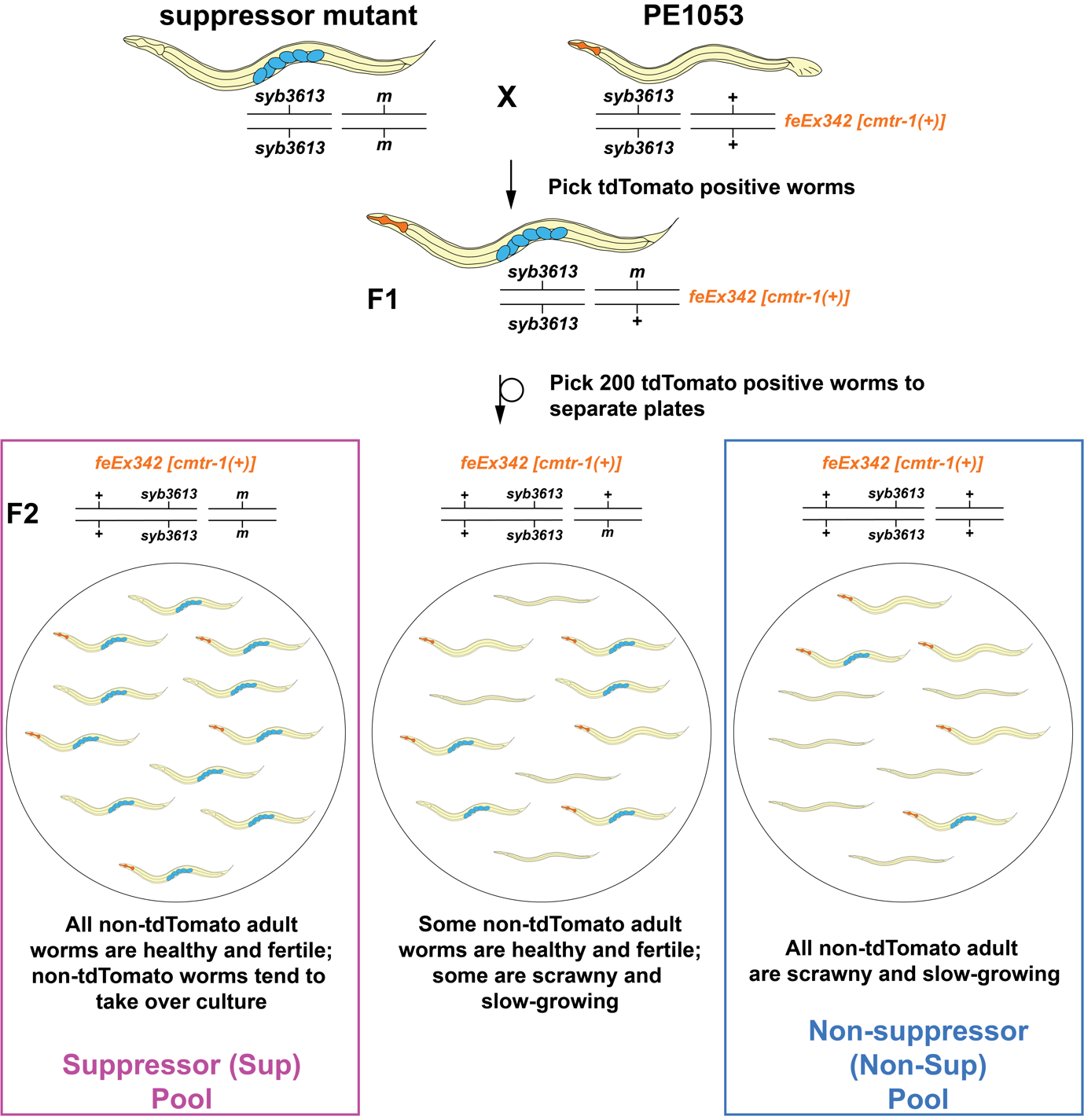
**

**Supplementary Figure 3. Strategy used to isolate strains for sibling-subtraction/whole genome sequencing mapping.** Suppressor strains were crossed to the transgenic rescue line, PE1053, which is homozygous for *cmtr-1(syb3613)* but rescued by the *feEx342 cmtr-1(+)* transgene. The Sup and Non-Sup lines were established from single F2 animals on the basis that F2 suppressor homozygotes were not dependent on the *feEx342 cmtr-1(+)* transgene, while the broods of non-suppressor homozygotes resembled those of the PE1053 grandparent strain. Note, *feEx342* shows relatively poor rescue of the *cmtr-1* loss-of-function phenotype, so some transgenic animals are sterile. Transgenic animals were recognised based on the *myo-2p::tdTomato* expression in the pharynx, which is also carried on the *feEx342* extrachromosomal transgenic array. Blue ovals indicate eggs inside gravid hermaphrodites.
